# A natural language processing–driven map of the aging research landscape

**DOI:** 10.18632/aging.206340

**Published:** 2025-11-25

**Authors:** Jose Perez-Maletzki, Jorge Sanz-Ros

**Affiliations:** 1 Universidad Europea de Valencia, Faculty of Health Sciences, Department of Physiotherapy, Nutrition and Sports Sciences, Valencia 46010, España; 2 Group of Physical Therapy in the Ageing Process: Social and Health Care Strategies, Department of Physical Therapy, Universitat de València, Valencia 46010, Spain; 3 Department of Pathology, Stanford University School of Medicine, Stanford, CA 94305, USA

**Keywords:** aging, literature mining, natural language processing, topic modelling, synthesis

## Abstract

Aging research has advanced significantly over the past century, from early studies on animal models to a current emphasis on clinical and translational applications. As research literature expands exponentially, traditional narrative reviews can no longer capture the field’s complexity, highlighting the need for new, unbiased synthesis tools. Here, we leverage advanced natural language processing (NLP) and machine learning (ML) techniques to analyze 461,789 abstracts related to aging published between 1925 and 2023. By integrating Latent Dirichlet Allocation (LDA), term frequency-inverse document frequency (TF-IDF) analysis, dimensionality reduction and clustering, we delineate a comprehensive thematic landscape of aging research. Our results show a clear shift: early decades focused on cellular and molecular mechanisms, while recent years emphasize clinical studies, especially neurodegenerative disorders. Notably, we identify a persistent divide between the biology of aging (BoA) and clinical research, with minimal conceptual overlap between them. Furthermore, we identify distinct clusters representing key biological processes, some of which may have previously been overlooked as cohesive research domains. Finally, we highlight both established and underexplored interconnections that could guide future research. This study outlines shifting priorities and translational gaps in aging research and offers a scalable, data-driven alternative to conventional reviews.

## INTRODUCTION

Early discoveries in the 20th century showed that the longevity of research animals could be extended by external factors such as caloric restriction [1, 2], and that mutations in specific genes, including *age-1* from the insulin/IGF-1 signaling pathway, can significantly increase lifespan [3, 4]. These studies led to the assumption that aging is a modifiable process, creating a whole new scientific field. In the last decades, aging research has grown exponentially, revealing multitude of genes and signaling pathways regulating the aging process, as well as many interventions that increase lifespan and health span in animal models [5]. This growth in the number of research articles describing processes related to the BoA has driven several attempts to summarize the field [6–8]. These reviews of the literature have been tremendously helpful to focus on aspects of aging biology with the highest scientific evidence. However, while they have greatly influenced the field, they may have inadvertently concentrated research efforts on specific topics, leaving other emerging trends underexplored. As research becomes more focused on these subfields, the emergence of new narratives in aging research is becoming more challenging. Moreover, the field is now being more populated by trends in healthcare and clinical studies, which have started to focus on aging as a key clinical variable and how it affects disease risk and progression. Yet the interaction between the BoA and clinical practice remains at a very premature stage.

When trying to accomplish a comprehensive literature review, authors are always biased towards personal preferences and knowledge about that field. With the exponential rise in the number of published research articles related to aging, it is becoming increasingly complex to summarize the whole field. Therefore, we believe that analyzing scientific literature from an unbiased perspective using natural language processing (NLP) computational tools represents a powerful alternative to narrative or systematic reviews. Our goal in this study is to provide a comprehensive, data-driven synthesis of the aging research landscape. By leveraging large-scale computational approaches, we aim to uncover thematic structures, research trends, and translational gaps that are difficult to capture through traditional experimental or review-based methods.

Here we leveraged different NLP techniques and machine learning (ML) models to perform an unsupervised analysis of a dataset comprising all available abstracts in the PubMed database (1925-2023) that include the term “aging” (~500,000). By using topic modeling and TF-IDF analysis [9], we revealed the most dominant topics in the field, their relationships with other topics and how they have evolved in the last 50 years. This analysis showed that while in the early years aging research focused on animal models, in the most recent years, clinical research and healthcare have emerged as dominant topics incorporating aging as a variable. Looking into specific tissues or systems, the diseases from the central nervous system (CNS), mainly Alzheimer’s disease (AD) and dementia, have concentrated research efforts, probably influenced by funding policies.

Inspired by recent tools developed for single-cell RNA-seq analysis [10], we used dimensionality reduction and clustering techniques to better understand how the manuscripts related to aging are distributed. To this end, we employed text vectorization to represent each abstract as a vector in a high-dimensional space, followed by Uniform Manifold Approximation and Projection (UMAP) embedding [11] and Leiden clustering [12]. This approach allowed us to extract a multitude of clusters that comprise different subfields in aging research, from healthcare to molecular biology. As one of the most prominent branches of aging research, we conducted a focused analysis on documents related to the BoA, being able to identify different clusters of manuscripts that encompass important themes in aging, including cellular senescence, telomeres or oxidative stress. By integrating the clustering information, the terms contained in each document, and the year of publication, we mapped the evolution of these clusters and how they are related to each other.

Looking into specific terms, we studied the distribution of the hallmarks of aging [7] in these clusters, extracting valuable information about how the hallmarks are distributed in our unbiased BoA clusters. This analysis revealed that certain hallmarks of aging are confined to more isolated clusters of documents, such as those focusing on telomeres, autophagy, or mitochondria. In contrast, others, such as inflammation or metabolism, are more evenly distributed through the literature, indicating a higher degree of interconnection with other aspects of aging research. Notably, this analysis allowed us to uncover overlooked relationships in the BoA literature, such as the connections between metabolism and telomeres or autophagy and epigenetics. To broaden the scope of this analysis and find underrepresented relationships between the different subfields of aging research, both from a global perspective and BoA-focused, we examined the presence of the top differential words from each cluster in every other cluster. This strategy unveiled the most extensively studied relationships and uncovered the least explored connections in the field, supporting the conclusion that the integration between BoA and clinical research remains immature and highlighting potential directions for future investigation. Overall, our analysis is a valuable resource for studying and tracing the evolution of key topics and themes in the aging field, identifying research opportunities, and serving as a versatile and scalable tool that streamlines literature analysis from an unbiased perspective.

## RESULTS

### Topic modeling reveals key topics, trends and interconnections in aging research

**Figure 1:**
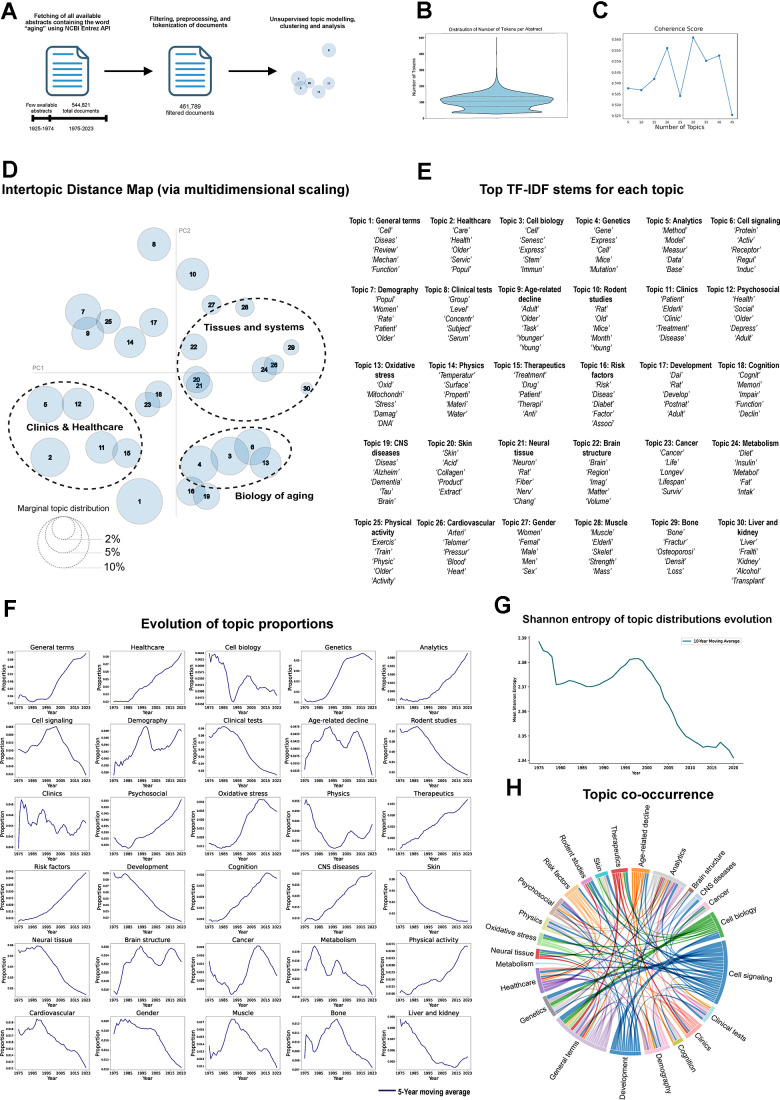
**Topic modeling of aging related abstracts.** (**A**) Workflow. (**B**) Distribution of number of words per abstract. (**C**) Coherence score of different number of topics using LDA topic modeling. (**D**) Intertopic Distance Map (PCA) of topics identified through LDA, distance between bubbles corresponds to the similarity between topics and the size represents the proportion of each topic within the corpus. (**E**) Top differential stems of each topic based on TF-IDF scoring. (**F**) Evolution of topic distributions along the whole corpus, each line represents the 5-year moving average of each topic proportions (1975-2023). (**G**) Shannon entropy evolution of topics distribution (1980-2023). (**H**) Chord diagram illustrating the co-occurrence of topics in the document corpus. The width of each chord is proportional to the co-occurrence magnitude.

Topic modeling has been widely applied across various domains, including social media analysis and market research, to extract abstract topics from textual datasets [13, 14]. In this study, we leveraged NLP and ML techniques commonly used in topic modeling to identify and analyze the evolution and interrelationships of key topics in aging research literature from an unbiased perspective. To achieve this, we retrieved the abstract text and publication year of all manuscripts containing the term “aging” or its variants in the abstract, title, or keywords using the NCBI Entrez API in the PubMed database. This process yielded 544,821 unique abstracts, with a limited number of abstracts available before 1975, which were subjected to tokenization and filtering steps, resulting in a final dataset of 461,789 unique abstracts with a median token count of 100 per abstract (Figure 1A, 1B).

For topic modeling, we employed LDA, a generative probabilistic model widely used in NLP [13], designed to uncover hidden thematic structures (topics) within large corpus of textual data. LDA assumes that documents are mixtures of multiple topics, with each topic characterized by a probability distribution over words. To determine the optimal number of topics for training, we calculated the coherence score by iteratively training an LDA model with varying numbers of topics. This analysis identified 30 topics as the most coherent number (Figure 1C). Subsequently, we trained an LDA model to extract topics from the documents and analyzed the most distinctive words within each topic using TF-IDF scoring [9]. This statistical measure highlights the significance of a word in a specific document relative to a collection of documents. Notably, we identified 30 well-defined topics encompassing key concepts relevant to the field, including clinics and healthcare, cell and molecular biology, and specific tissues such as brain or muscle (Figure 1D and Supplementary Data 1).

Among these, a topic containing general terms emerged as the most prevalent across the dataset, followed by topics related to healthcare and cell biology (Figure 1E and Supplementary Table 1). Regarding tissue-specific topics, the aging field is prominently represented by terms associated with the central nervous system (CNS) and associated neurodegenerative diseases, with four distinct topics linked to this system. Other tissues prominently represented in the model include skin, cardiovascular, muscle, bone, liver, and kidney. However, some systems, including the digestive, respiratory, and reproductive systems, seem to be less studied in the aging field, as the analysis lacks specific topics related to them.

To analyze the temporal evolution of these topics over the last 50 years, we quantified the yearly topic proportions to identify trends for each topic (Figure 1F). The aging field appears to have transitioned from a predominantly biology-focused domain, characterized in earlier years by a higher proportion of topics related to cell biology, cell signaling, rodent studies or specific tissues, to a more clinically and human-oriented perspective. This shift is evidenced by an increased prominence of topics such as healthcare, analytics, demography, psychosocial aspects, therapeutics, risk factors, and physical activity in recent years. Interestingly, CNS is the only tissue-specific topic that exhibits a positive trend over time.

To assess whether the field has become more varied or more concentrated in its focus over the years, we calculated the evolution of Shannon entropy of topic distributions (Figure 1G). Shannon entropy quantifies uncertainty in a distribution, with higher values reflecting greater diversity and lower values indicating a focus on fewer dominant themes. The observed decrease in Shannon entropy of topic distributions over time suggests a narrowing diversity in the topics being studied, pointing to a shift towards convergence within the field. Concurrently, the increasing prevalence of general terms may reflect the rise of more generalist manuscripts, likely influenced by the growing number of secondary research outputs such as narrative or systematic reviews and meta-analyses relative to original research [15, 16]. This could suggest field maturation, with researchers consolidating knowledge and building on established frameworks. However, it could also be interpreted as a sign of reduced diversity, with fewer novel ideas and a potential narrowing of intellectual breadth, a recently raised concern across many scientific fields [17].

To uncover the interconnections between topics, we analyzed their co-occurrence within the documents in the dataset (Figure 1H and Supplementary Figure 1). Unsurprisingly, broader topics like general terms, analytics, cell biology, and cell signaling emerged as central hubs, linking to a wide range of other topics. However, a clear pattern emerged: topics related to clinics and healthcare, such as therapeutics, psychosocial aspects, and risk factors, frequently appear together, while biology-focused topics show a similar tendency to co-appear. This division highlights the distinct focus areas within the aging field, with biological mechanisms and healthcare-related perspectives largely developing in parallel rather than being closely interconnected. As a final step, we performed a sentiment analysis of the documents and examined how it evolved over time. Words with positive sentiment included “strength,” “care,” “training,” and “healthy,” whereas terms like “cancer,” “depression,” and “damage” scored negatively, aligning with their intuitive meanings (Supplementary Figure 2A). Overall, sentiment remained positive throughout the analyzed period, rising in the initial years but starting to decline around the early 2000s, when research on the link between cancer and aging intensified (Supplementary Figure 2B, 2C). Assessing the sentiment associated with each topic showed that “muscle” and “healthcare” exhibited the most positive sentiment, whereas “oxidative stress” and “cancer” were the only topics displaying a negative sentiment score (Supplementary Figure 2D).

These findings reveal clear trends in the aging field, including a shift from biological mechanisms toward healthcare and clinical perspectives, alongside a narrowing diversity in topics. The dominance of CNS-related topics highlights the central role of neurodegenerative diseases in aging research. Additionally, the separate grouping of biological and healthcare-related topics suggests parallel developments with limited integration.

### Mapping the thematic landscape in aging research through document vectorization and clustering

**Figure 2:**
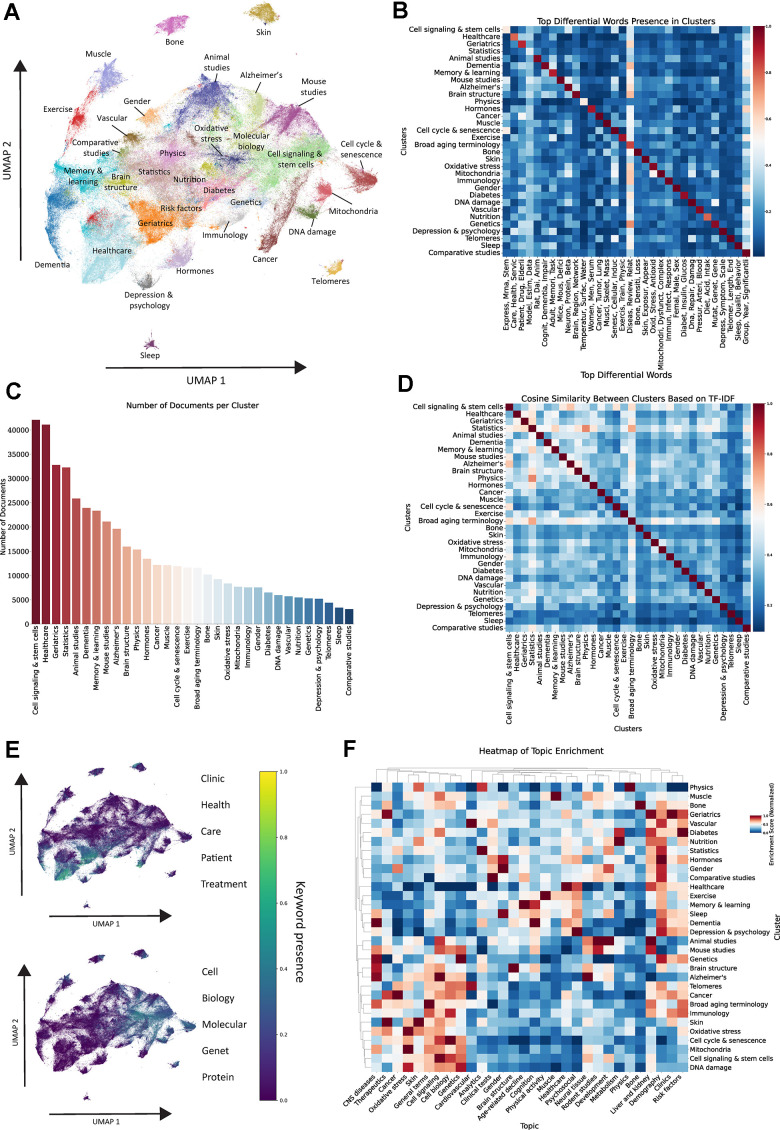
**Unsupervised clustering of aging related abstracts.** (**A**) UMAP and Leiden clustering of vectorized documents in the dataset. (**B**) Heatmap showcasing top differential words in each cluster, color reflects the proportion of documents within each cluster containing any stem from the respective word group. (**C**) Number of documents per cluster. (**D**) Cosine similarity analysis between clusters based on TF-IDF vectors. (**E**) UMAP representation highlighting the presence of keywords related to clinics and healthcare vs. molecular and cell biology. (**F**) Hierarchical clustering heatmap of topic enrichment in each cluster, color reflects normalized enrichment score.

Aging research spans a diverse range of domains, from molecular mechanisms to clinical applications. To better understand how these themes are structured, we applied dimensionality reduction techniques to examine the relationships between key topics in the field. Preprocessed documents were transformed into a high-dimensional vector using TF-IDF vectorization [9], which considers the importance of each term in a document relative to the entire corpus. To represent each document in a 2-dimensional space, we employed Principal Component Analysis (PCA) and UMAP [11] embedding (Figure 2A), followed by Leiden clustering [12], which revealed a well-defined clustering of documents in different themes that contain cluster-specific terms (Figure 2B and Supplementary Figure 3 and Supplementary Table 2). Our analysis identified clusters corresponding to well-established domains, including cell and molecular biology, such as “Mitochondria” and “DNA damage”, “Cell cycle and senescence”, and “Oxidative stress”; clinical and healthcare-related clusters, such as “Healthcare”, “Depression and psychology”, and “Geriatrics”; and lifestyle and demographic factors, including “Exercise”, “Nutrition”, and “Risk factors”. Additionally, we also found clusters related to specific tissues and systems, such as, “Muscle”, “Bone”, and “Skin”, which tended to be well-separated from the rest of the corpus. The distribution of documents across these clusters (Figure 2C) revealed that clusters containing broader terms, such as “Healthcare” and “Cell signaling and stem cells”, encompass a larger number of documents. In contrast, clusters containing more specialized terms, such as “Telomeres” and “Sleep”, exhibit smaller cluster sizes.

To quantify the relationships between our clusters, we computed cosine similarity (Figure 2D), which measures their semantic proximity within the embedding space by averaging the TF-IDF vectors of all documents within each cluster. Pairs with higher cosine similarity, such as “Cell cycle and senescence” and “Cell signaling and stem cells” or “Diabetes” and “Nutrition”, indicate closely related research areas with significant conceptual overlap. In contrast, clusters such as “Skin”, “Muscle” or “Telomeres”, exhibit lower similarity scores with other clusters, highlighting their distinct focus and minimal conceptual intersection with the rest of the corpus.

This analysis revealed a clear distinction between clinical and healthcare-related terms and those associated with the BoA within the 2D space (Figure 2E). Clinical and healthcare-related terms, such as “Healthcare”, “Patient”, and “Treatment”, are predominantly positioned in one region of the space (left), whereas biology-related terms, including “Cell”, “Molecular”, and “Protein”, cluster in a distinct and separate region (right).

To integrate both topic modeling and clustering, we employed hierarchical clustering based on topic enrichment in each cluster (Figure 2F). This approach showed that clusters are generally highly enriched in topics that closely align with their defining terms. For example, the “Healthcare” cluster is predominantly associated with the healthcare-related topic, while the “Exercise” cluster exhibits strong enrichment for the physical activity-related topic, indicating a consistent thematic alignment across the dataset. Through this approach, we identified three distinct hierarchical groups within the clusters. The first hierarchy consists of clusters related to the BoA, which are primarily enriched with topics focused on cell and molecular biology (“DNA damage”, “Oxidative stress”, “Mitochondria”, etc). The second hierarchy includes healthcare and clinical clusters, which are predominantly associated with clinically relevant topics (“Healthcare”, “Dementia”, “Depression and psychology, etc). The third hierarchy comprises tissue-specific clusters, including “Muscle”, “Bone”, and “Vascular”, which display a more diverse and sparse enrichment pattern across different topics. An interesting observation emerges when comparing the “Muscle” and “Exercise” clusters. While both are related to physical activity and musculoskeletal health, the “Muscle” cluster shows greater enrichment in topics associated with BoA. In contrast, the “Exercise” cluster is more aligned with lifestyle and clinical-related topics, highlighting the dual focus within musculoskeletal aging research.

Our approach highlights clear thematic organization in well-defined clusters comprising different aspects of aging research, ranging from healthcare, clinical and social domains to fundamental biology. The relative scarcity of documents containing healthcare-related terms within biology-focused clusters, and vice versa, underscores the persistent divide between basic and clinical research. This pattern suggests that, despite growing interdisciplinary efforts, these domains largely remain separate in focus and scope.

### Temporal analysis of aging research clusters

**Figure 3:**
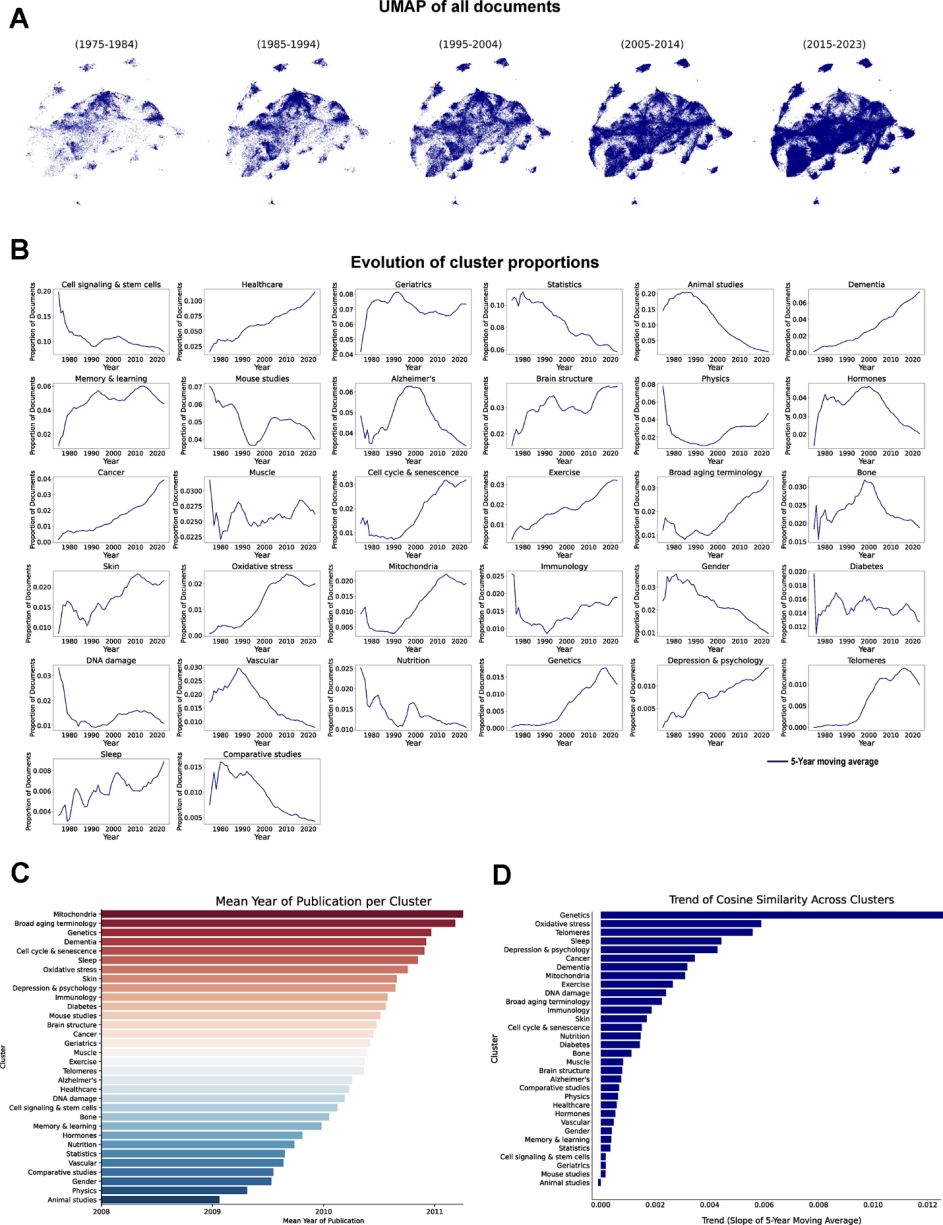
**Temporal evolution of aging research clusters.** (**A**) UMAP of vectorized documents distributed across decades. (**B**) Evolution of cluster proportions, each line represents the 5-year moving average of each cluster proportion (1975-2023). (**C**) Mean publication year of the documents in each cluster. (**D**) Cosine similarity trend analysis across clusters, results represent the slope of the 5-year moving average of the changes in cosine similarity within each cluster (1975-2023).

After characterizing the thematic composition of the clusters, we analyzed their temporal evolution to identify distinct trends reflecting shifting research priorities within the aging field. As expected from the steady growth in aging-related manuscripts over the years, we observed an overall increase in the number of published papers across nearly all clusters (Figure 3A). However, despite this general growth, the field has consistently shifted towards a stronger emphasis on clinical aspects (Figure 3B). Clusters related to “Healthcare”, “Dementia”, and “Cancer” have gained prominence, reflecting the increasing focus on aging as a key variable in clinical research. This shift occurred at the expense of clusters primarily focused on the BoA, which has seen a relative decline in their representation within the field. Notable exceptions to this trend include the “Oxidative stress”, “Cell cycle and senescence”, and “Genetics” clusters, which have increased over time. This trend is particularly evident in clusters related to CNS. While the proportion of papers related to “Dementia” or “Memory and learning”, clinically oriented clusters, has consistently increased, clusters such as “Alzheimer’s”, which have a clear focus on more fundamental aspects, have shown a declining trend from the early 2000s.

We then calculated the mean publication year of papers within each cluster (Figure 3C) to examine which areas have gained prevalence in the last years, revealing that clusters such as “Mitochondria” and “Genetics” emerge as the most recent. The trajectory of the clusters related to animal models is particularly noteworthy. “Animal studies”, which represents the oldest area of research and is the only cluster where the absolute number of publications has declined from the early 2000s, contains mainly studies using rat as the animal model. Meanwhile, the “Mouse studies” cluster had a later development, showcasing the transition from rat-based models, which historically dominated earlier studies, to the increasing use of mice as the standard model in aging research.

To further explore the stability and evolution of thematic focus within clusters, we analyzed the trends in temporal consistency of topic distributions using cosine similarity (Figure 3D). This analysis quantifies the degree of thematic consistency over time by calculating the similarity of topic distributions between consecutive years within each cluster. A steep positive trend indicates a rapid increase in thematic stability, suggesting that the cluster converged early and has maintained a consistent focus over the years. In contrast, clusters with a negative trend demonstrate greater variability, reflecting ongoing thematic shifts and relatively higher introduction of new concepts within those research areas. Overall, we observed an increase in cosine similarity across most clusters, indicating a trend towards greater thematic consolidation within the aging research field. Notably, clusters such as “Genetics”, “Oxidative stress” and “Telomeres” exhibit the steepest positive trends, implying earlier convergence and sustained thematic focus. Conversely, only the “Animal studies” cluster show a negative trend, suggesting a change in topic distribution over time.

A clear evolution in research priorities emerges from our findings, indicating a growing emphasis on clinical and healthcare-related clusters at the expense of BoA-focused clusters. However, key areas in BoA research, such as oxidative stress and cellular senescence, continue to expand. Additionally, our analysis suggests that thematic shifts within aging research are becoming less frequent, with clusters converging towards more stable topic compositions.

### Focused analysis uncovers thematic patterns in biology of aging research

**Figure 4:**
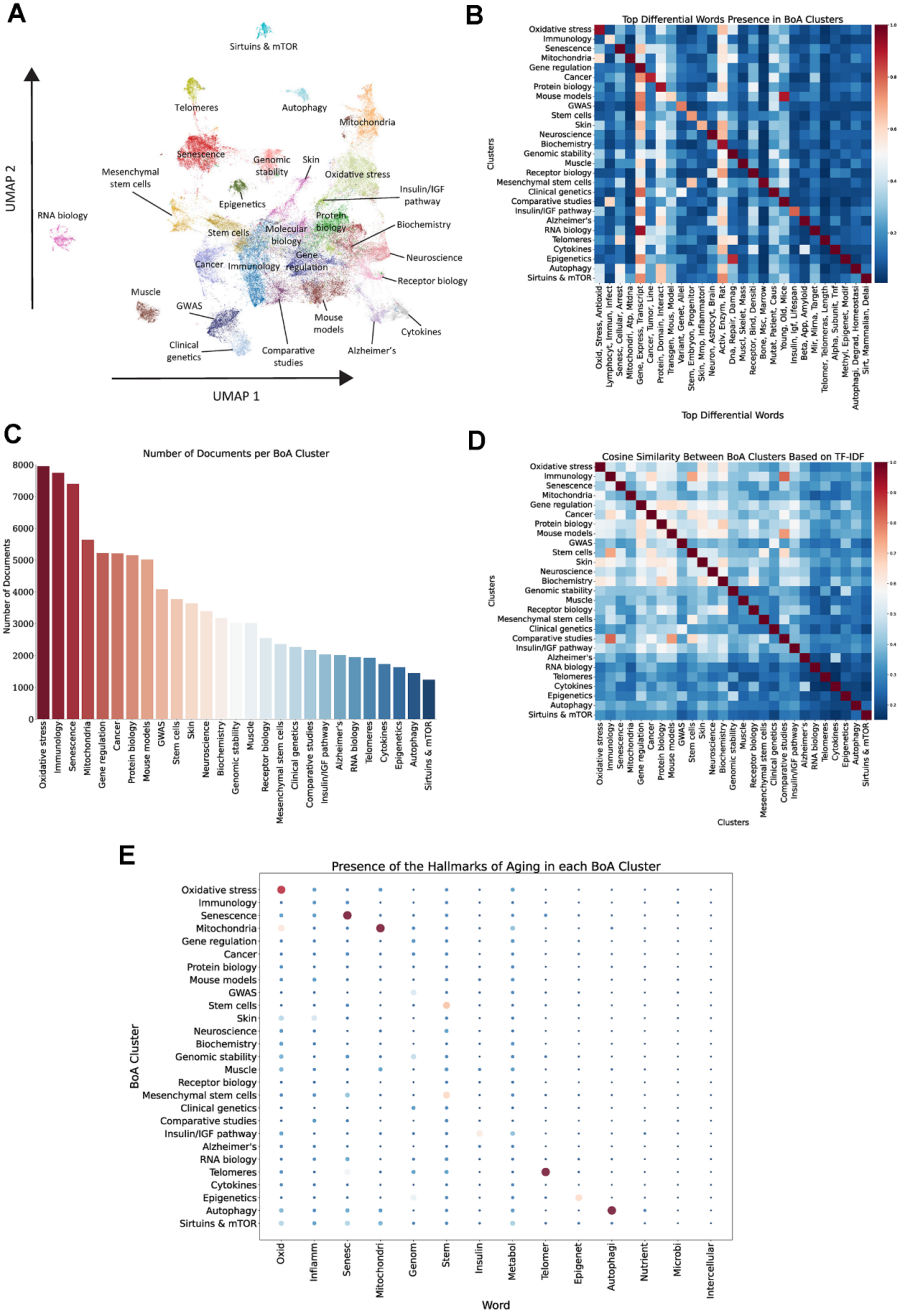
**Unsupervised clustering of BoA-related abstracts.** (**A**) UMAP and Leiden clustering of vectorized BoA-related documents. (**B**) Heatmap of top differential words in each cluster, color reflects the proportion of documents within each cluster containing any stem from the respective word group. (**C**) Number of documents per cluster. (**D**) Cosine similarity analysis between clusters based on TF-IDF vectors. (**E**) Dot plot of the relative presence of stems associated with the Hallmarks of Aging in each cluster, size and color of the dots represent the proportion of documents that contain a stem within a particular cluster.

To accomplish a more detailed analysis of one of the most influential branches of aging research, we extracted abstracts whose main topic corresponded to one of the BoA-related topics from topic modeling, yielding approximately 94,000 documents. Then, we applied again dimensionality reduction (PCA and UMAP) to the vectorized documents (Figure 4A), followed by Leiden clustering, uncovering several clusters of documents containing terms specific to different domains of the BoA (Figure 4B and Supplementary Figure 4 and Supplementary Table 3). This focused analysis identified clusters corresponding to well-established domains in the BoA field, including “Senescence”, “Oxidative stress”, “Mitochondria”, “Epigenetics”, “Autophagy” or “Telomeres”. Additionally, clusters associated with aging-related diseases (“Alzheimer’s” and “Cancer”), also emerged, emphasizing their prominence within BoA research. This likely reflects a more intensive focus on their underlying biological mechanisms compared to other age-related diseases. Furthermore, two well-separated tissue-specific clusters, “Muscle” and “Skin”, appeared, suggesting a distinct research focus on their fundamental biological processes within aging compared to other tissues.

The identified clusters exhibit notable differences in document prevalence (Figure 4C). Broader research domains, such as “Oxidative stress”, “Immunology”, and “Senescence”, are represented in the largest clusters, reflecting their centrality and wide coverage within the BoA. In contrast, more specialized or niche themes, including “Sirtuins and mTOR”, “Autophagy”, and “RNA biology”, contain fewer documents and are usually more separated in the 2D space, highlighting their more focused and specific areas of research.

Cosine similarity analysis (Figure 4D) revealed the conceptual relationships between clusters by measuring their semantic proximity. Stronger relationships emerged between clusters such as “Stem cells” and “Mesenchymal stem cells”, or “Epigenetics” and “Genomic stability”, indicating some thematic overlap. In contrast, clusters including “RNA biology”, “Autophagy”, and “Sirtuins and mTOR” displayed lower similarity scores with other clusters, underscoring their distinct thematic focus and limited conceptual intersection with broader research domains.

Finally, to further investigate the distribution of hallmark-associated terms across our clusters and evaluate how each cluster aligns with the hallmarks of aging, we calculated the presence of hallmark-related terms within each cluster (Figure 4E). This analysis revealed that certain hallmarks are strongly represented within specific clusters, while others are more widely distributed across multiple clusters. For example, terms related to “Oxidative stress”, “Senescence”, and “Mitochondria” are predominantly concentrated within their corresponding clusters, reflecting a clear thematic alignment. Similarly, the “Telomeres”, “Autophagy”, “Epigenetics” and stem cell-related clusters show a strong association with their respective hallmarks. In contrast, hallmarks such as “Genomic instability”, “Inflammation”, “Metabolism” and “Altered intercellular communication” are more diffusely represented across multiple clusters, suggesting they are likely studied from more diverse perspectives. Notably, cellular senescence, oxidative damage, genetics and metabolism are the most prevalent terms across the entire document corpus. This comparison highlights how certain clusters align closely with specific hallmarks of aging, while others exhibit a more diffuse distribution without a clear hallmark association.

A distinct and coherent thematic framework in BoA research becomes evident through our analysis, with clusters aligned to fundamental aging processes, diseases, and tissues. While some clusters closely correspond to individual hallmarks of aging, others do not map to a specific hallmark, suggesting that dividing the field into hallmarks does not fully encompass all areas of BoA research.

### Temporal dynamics in biology of aging research

**Figure 5:**
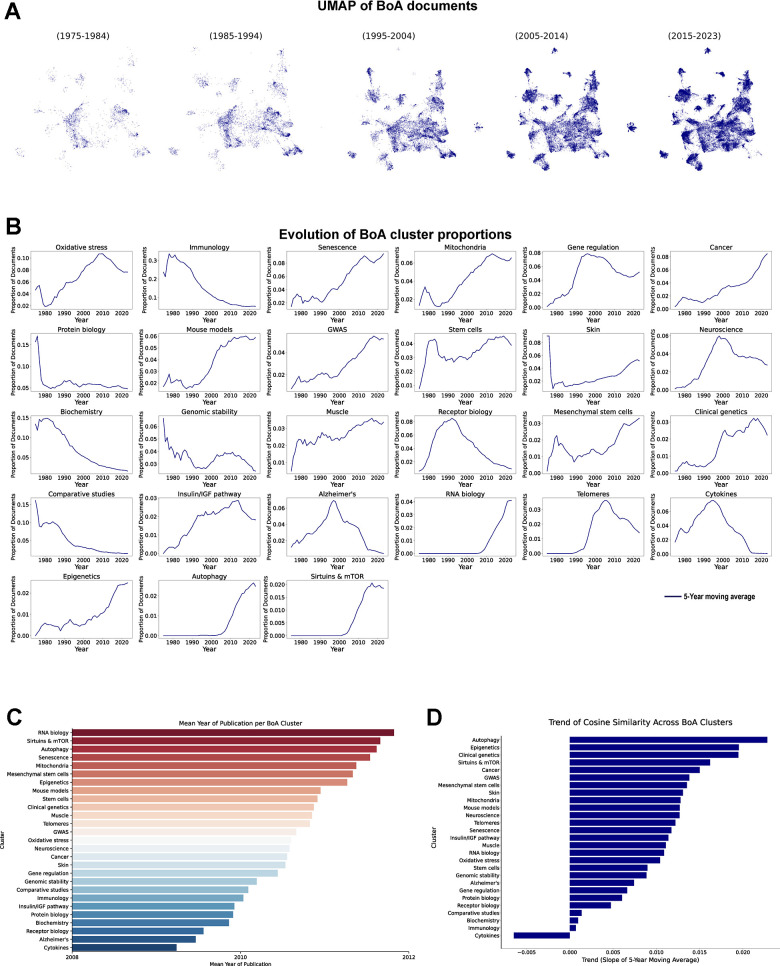
**Temporal evolution of BoA research clusters.** (**A**) UMAP of vectorized documents distributed in decades. (**B**) Evolution of BoA cluster proportions, each line represents the 5-year moving average of each cluster proportion (1975-2023). (**C**) Mean publication year of the documents in each cluster. (**D**) Cosine similarity trend analysis across clusters, results represent the slope of the 5-year moving average of the changes in cosine similarity within each cluster (1975-2023).

Following our assessment of the BoA clusters’ thematic composition, we examined their evolution over time to uncover shifts in research priorities. Consistent with the steady growth in aging-related manuscripts over the years, clusters generally showed an increase in the absolute number of published papers per year (Figure 5A).

Further analysis of the evolution of cluster proportions reveals a notable shift in research focus over time (Figure 5B). Specifically, more generalist clusters such as “Immunology” and “Biochemistry” have declined in their proportional representation. In contrast, dedicated clusters like “Oxidative stress”, “Senescence” or “Mitochondria” have expanded, reflecting that as the field has evolved, research has tended to specialize. Additionally, clusters that were absent in the early stages of the field, namely “Telomeres” “RNA biology” “Autophagy” and “Sirtuins and mTOR”, exhibited steep increases once their relevance to aging was established. The analysis of the mean publication year (Figure 5C) supports this trend, highlighting that these clusters are among the most recent additions to the field. Meanwhile, “Cytokines” and “Alzheimer’s” stand as the oldest clusters. This does not imply that these subfields are no longer actively studied, it rather suggests that manuscripts focusing primarily on these areas have become less common over time, potentially integrating into other clusters. An intriguing observation is that the “Alzheimer’s” cluster, even though Alzheimer’s disease is one of the most studied areas at the present time, exhibits a clear decline, probably because it has been integrated in more clinically oriented or translational research frameworks. Classical studies focusing on the molecular and biological underpinnings of AD, particularly in relation to aging, appear to have decreased in relative volume.

As previously implemented, we evaluated trends in the temporal consistency of topic distributions by applying cosine similarity (Figure 5D) to assess the evolution of thematic stability within each cluster. Overall, we observed an increasing trend towards thematic consolidation across most clusters within the BoA research field. Notably, clusters such as “Autophagy”, “Epigenetics”, and “Clinical genetics” exhibit the steepest positive trends, indicating rapid convergence and sustained thematic focus. In contrast, the “Cytokines” cluster is the only one to show a negative trend, suggesting a redistribution of scope during the evolution of this cluster.

The evolution of BoA research clusters over time reflects a reorientation of thematic focus. While the total number of BoA-related manuscripts has increased, broader categories such as “Biochemistry” and “Immunology” have diminished in relative proportion as research has become more specialized. Furthermore, assessment of temporal consistency indicates a clear trend toward convergence, with thematic shifts in each cluster becoming less frequent, consistent with the broader patterns observed when studying all documents.

### NLP–driven discovery of underexplored connections in aging research

Ultimately, we employed NLP to explore potential research gaps by focusing on the interactions between different clusters through semantic overlap analysis. We leveraged TF-IDF scoring to quantify the distribution of cluster-specific terms. For each cluster, we calculated the mean TF-IDF vector by averaging the TF-IDF scores of all its documents and then identified the top 20 distinctive words. Next, we measured how these top terms were represented in other clusters by averaging their TF-IDF scores across clusters. This approach enabled us to highlight over- and underrepresented relationships between clusters, revealing the most and least studied connections between subfields of aging research.

**Figure 6:**
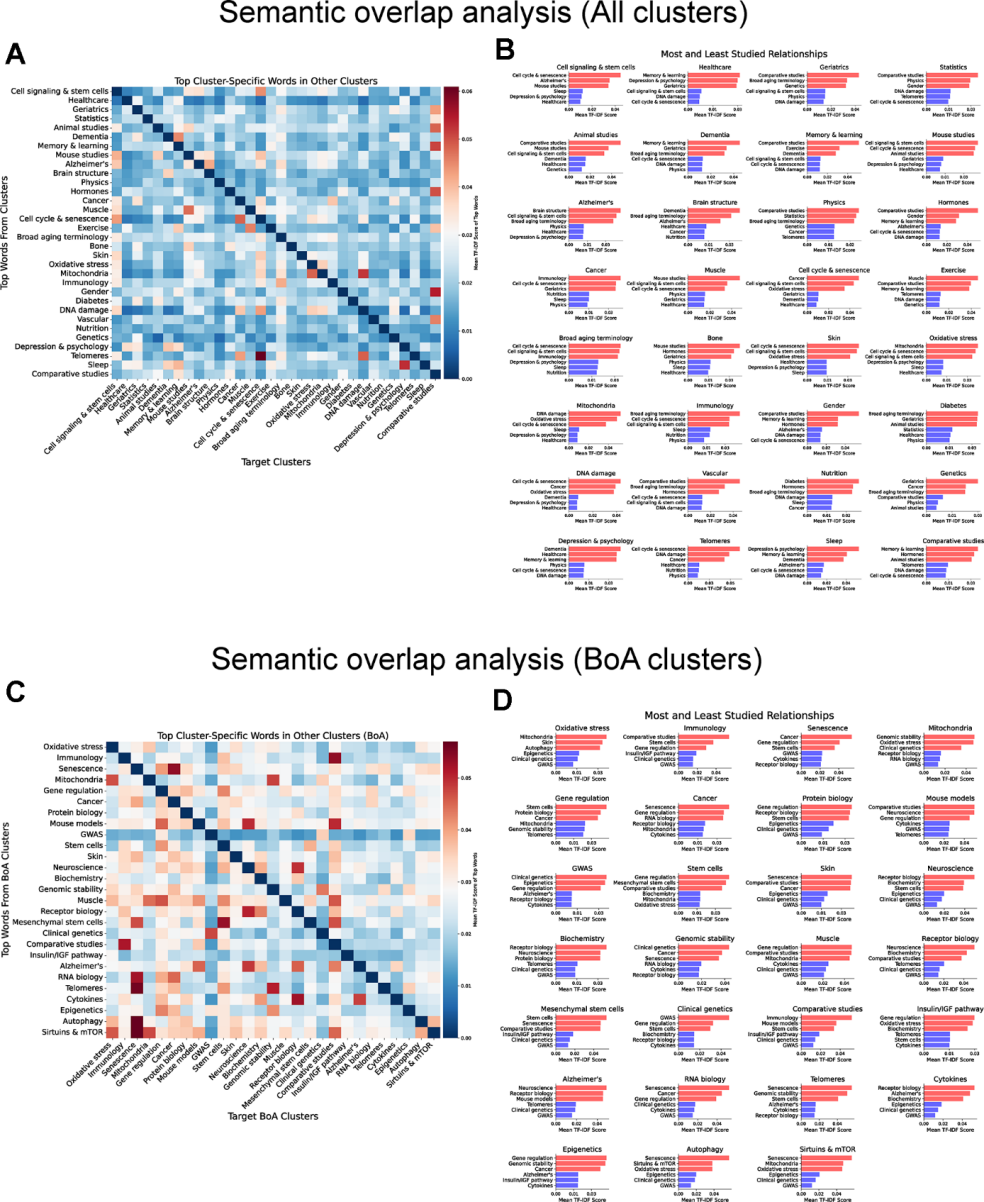
**Mapping underexplored connections in the aging research literature through semantic overlap analysis.** (**A**) Heatmap of average TF-IDF score of the top 20 most significant words from each cluster when evaluated against documents in every other cluster using the dataset containing all documents. Rows represent the source clusters from which the top 20 words were selected based on their TF-IDF score. Columns represent the target clusters where the mean TF-IDF scores of these words were computed. Color represents the magnitude of the average TF-IDF score. (**B**) Top 3 most and least studied relationships among clusters (all documents). (**C**) Heatmap of average TF-IDF score of the top 20 most significant words from each BoA cluster when evaluated against documents in every other BoA cluster using the dataset containing only BoA-related clusters. Rows represent the source clusters from which the top 20 words were selected based on their TF-IDF score. Columns represent the target clusters where the mean TF-IDF scores of these words were computed. Color represents the magnitude of the average TF-IDF score. (**D**) Top 3 most and least studied relationships among BoA clusters.

First, we analyzed the dataset containing all documents (Figure 6A, 6B). In general, clusters with thematically similar research focuses exhibited a higher number of relationships. For example, clinical domains such as “Healthcare” and “Geriatrics”, “Muscle” and “Exercise”, as well as “Memory and learning” and “Dementia”, displayed a broader range of connectivity. In contrast, the least studied relationships are often found between clinical-focused clusters and biology-focused clusters. For instance, central biological processes, such as “Cell cycle and senescence”, “Oxidative stress”, or “Mitochondria”, show limited exploration in connection with clinical topics such as “Healthcare” or “Geriatrics”. This pattern highlights a research gap, suggesting that fundamental biological processes are insufficiently connected with clinical outcomes, in line with previous results discussed in this manuscript.

Then, we examined relationships in the dataset focused on BoA clusters (Figure 6C, 6D). As expected, certain relationships are well-studied, reflecting their established importance in aging biology. For example, “Senescence” and “Cancer”, a central axis in age-related diseases, shows a relative over-representation. Similarly, “Mitochondria” and “Oxidative stress”, a cornerstone of aging theories, exhibit strong research connections. Other well-explored relationships include “Gene regulation” and “Epigenetics” and “Cancer” and “Senescence”, reflecting a tighter interconnection between these fields.

In contrast, the connection between several fundamental aging processes remains underexplored, revealing opportunities for new discoveries. For instance, despite their shared roles in cellular aging, “Senescence” and “Mitochondria” show limited research overlap. Similarly, “Oxidative stress” and “Epigenetics”, two key players in cellular damage and aging, remain poorly connected. Other notable examples of under-represented relationships include “Telomeres” and “Alzheimer’s” and “Autophagy” and “Epigenetics”.

Together, these findings underscore the opportunity for more integrative, cross-disciplinary research approaches to bridge biological mechanisms with clinical contexts. Focusing on the BoA clusters, our analysis reveals both well-explored areas and significant gaps in the interconnections between fundamental aging processes, highlighting key opportunities for future research into aging mechanisms.

## DISCUSSION

This study represents, to our knowledge, the first large-scale, unbiased computational analysis of aging research literature spanning nearly a century. By leveraging advanced natural language processing (NLP) and machine learning (ML) techniques on over 460,000 PubMed abstracts dating from 1925 to 2023, we have delineated the evolving thematic landscape of aging research. Our integrated approach combining topic modeling, TF-IDF weighting, dimensionality reduction, and clustering, provides a comprehensive map of the field, uncovering both well-established domains and emerging subfields. Our work not only charts historical shifts from fundamental biological investigations using animal models to a predominantly clinical and translational focus but also reveals underexplored interconnections between fundamental aging processes.

A central finding of our study is the marked evolution in research priorities over the past 50 years. Early decades were dominated by a focus on animal models and cellular mechanisms, which laid the groundwork for our mechanistic understanding of aging. In contrast, recent decades show a pronounced shift toward clinical research and healthcare applications, reflecting both technological advances and changing societal priorities as populations age. This transition is further underscored by our temporal analysis of topic proportions and Shannon entropy, which suggest a consolidation of research themes around a few dominant topics; most notably, those related to healthcare and clinics, and an intensive emphasis on neurodegenerative diseases where AD and dementia have emerged as the most studied conditions in the aging field. Such convergence, while indicative of maturation within the field, may also signal a narrowing of intellectual diversity that could constrain novel discoveries in less-explored areas. Importantly, the overwhelming dominance of AD and dementia research may not solely reflect emerging scientific trends but could also be partially driven by funding policies. For instance, agencies like the National Institute on Aging have historically allocated a substantial proportion of their research funding to Alzheimer’s and related dementias, shaping the field’s research priorities.

Our clustering analysis revealed distinct thematic groups that not only segregate clinical and basic biological research but also highlight specific tissue- and system-focused studies (e.g., those related to the CNS or skeletal muscle). Links between BoA clusters (such as oxidative stress and cellular senescence) and clinically oriented clusters remain sparse. This suggests that despite the overall growth in aging research, a significant gap persists between fundamental aging mechanisms and their translation to clinical settings. Addressing this gap could open new avenues for integrative, cross-disciplinary research and ultimately improve patient outcomes.

Beyond differences in funding distribution, several structural and conceptual barriers contribute to the persistence of this gap. These include disciplinary silos that limit collaboration between basic aging biologists and clinical researchers, the absence of shared conceptual frameworks and standardized vocabularies linking molecular hallmarks of aging with clinical phenotypes, and methodological mismatches in experimental design, model systems, and outcome measures [8, 18]. Together, these factors can hinder the integration of fundamental biological insights into clinical practice and slow the development of translational interventions.

Building on these observations, we propose several strategies to bridge this divide and foster a more integrated research landscape. One key step is the development of interoperable ontologies that explicitly map molecular and cellular aging processes to clinical outcomes, aligning experimental models with human health endpoints. The creation of standardized terminologies and shared conceptual frameworks across basic and clinical domains would further unify vocabularies and enable cross-disciplinary data integration. In addition, cross-disciplinary consortia and collaborative initiatives that bring together geroscientists, clinicians, computational biologists, and epidemiologists could help break down institutional and conceptual barriers, promoting dialogue and shared research priorities.

We also highlight the need for open, multimodal datasets that link biological markers, preclinical models, and patient-level clinical data, providing a foundation for robust translational studies. Prospective longitudinal cohort studies incorporating molecular aging biomarkers alongside clinical outcomes could generate the critical evidence needed to validate mechanistic insights in human contexts. Moreover, incorporating geroscience endpoints into ongoing clinical trials for age-related diseases could accelerate translation by linking biological hallmarks to therapeutic responses. Together, these strategies would support a more cohesive and bidirectional research ecosystem, accelerating the translation of fundamental discoveries into interventions that improve human healthspan.

Beyond these broad trends, a focused analysis on the BoA research literature uncovered distinct clusters corresponding to fundamental aging processes. When we compared these clusters with the well-established hallmarks of aging, we found that while some clusters align closely with these predefined categories, others do not clearly fit into them. This discrepancy suggests that the BoA is more diverse than what the classical hallmarks scheme alone might capture. This comparison underscores the value of our unbiased, data-driven approach in revealing research themes beyond predefined frameworks.

In addition to mapping research trends, our analysis uncovered several underrepresented or overlooked relationships in the aging field. By quantifying the differential presence of cluster-associated terms, we outlined areas where potential synergies remain underexplored. For example, clusters related to clinical domains such as “Healthcare” and “Geriatrics”, or “Muscle” and “Exercise”, demonstrated considerable vocabulary overlap, reflecting well-established connections in applied research. In contrast, clusters representing biological processes, such as those involved in the cell cycle, oxidative stress, and mitochondrial function showed limited intersections with clinical topics. Notably, while certain links between fundamental BoA processes, such as “Senescence” and “Cancer” or “Mitochondria” and “Oxidative stress”, are extensively studied, our data reveal that other potentially critical relationships, for instance “Senescence” and “Mitochondria” or “Autophagy” and “Epigenetics,” remain underexplored. These insights offer a powerful roadmap for the aging research community to identify and investigate underexplored relationships that may hold potential for advancing the field.

Traditional narrative or systematic reviews have invariably been limited by subjective biases and the growing volume of scientific literature. Our unsupervised, data-driven approach circumvents these limitations, offering an unbiased synthesis of research trends that is highly scalable and adaptable, with potential applications beyond aging research.

Nevertheless, our study is not without limitations. One key limitation is the abstraction-level bias inherent in our dataset: the analysis is based on article abstracts rather than full texts, which may lead to a loss of nuance and contextual information. Abstracts provide concise summaries and are suitable for large-scale topic modeling, but they do not capture the complete depth of the underlying studies. This limitation may influence topic representation and should be considered when interpreting our results. An additional limitation of this study arises from potential biases introduced by PubMed’s indexing practices. The scope, structure, and curation policies of the database influence which studies are included, how they are categorized, and how searchable metadata are assigned. These factors may shape the thematic patterns captured by our analysis, potentially emphasizing certain types of research or publication formats over others. Our analysis is shaped by the conceptual and technical characteristics of the LDA and TF-IDF framework. These methods make some simplifying assumptions, such as topic independence and the conditional independence of words within topics, which, while effective for capturing broad thematic structures, may limit the ability to fully reflect polysemy, semantic nuance, or hierarchical relationships among topics. More advanced transformer-based language models could potentially address some of these challenges by capturing richer contextual relationships, but their application at this scale and for this type of bibliometric analysis remains computationally intensive and recent advances now make their use in bibliometric analyses increasingly feasible, representing a promising direction for future work [19, 20].

It is important to note that the topic and cluster labels presented in the manuscript are intended to enhance the interpretability and readability of the results; they are not claimed to represent definitive or exhaustive categorizations of the research landscape. To promote transparency and enable independent interpretation, we provide the full list of TF-IDF-derived top words for each topic and cluster, as well as an interactive LDA visualization. This resource will allow readers to explore topic identities, thematic relationships, and interpretations based on their specific research questions.

In summary, our study provides a transformative perspective on aging research by systematically mapping its historical evolution, elucidating complex thematic interrelationships, and identifying research gaps. These findings may serve as a catalyst for further interdisciplinary collaboration and the development of innovative strategies to bridge the gap between fundamental aging research and clinical application, while also laying a scalable foundation for future studies leveraging emerging NLP methods and expanded datasets.

## MATERIALS AND METHODS

### Abstract fetching and data preprocessing

To generate the dataset, we used the Entrez API through the Biopython library [21] and searched for the manuscripts that contained the word “aging” in the abstract, title, or keywords from 1925 to 2023. For each manuscript, title, abstract and year of publication were retrieved. A function to remove duplicates was applied to the dataset. The corpus was retrieved using English search terms, which naturally yields an English-based dataset. Although no explicit language filter was applied during preprocessing, a post hoc language analysis using the langid Python library identified only 172 non-English records across the entire dataset. Abstracts were tokenized using the preprocess_string function from Gensim library [22], breaking each abstract into individual tokens and applying standard preprocessing steps, including lowercasing, removal of punctuation, and elimination of common stop words (listed in Supplementary Table 4). To ensure enough semantic content for topic inference while minimizing noise from atypical documents, only abstracts containing between 30 and 500 tokens were retained. Abstracts shorter than 30 tokens typically lacked enough contextual information to support stable topic modeling, while those exceeding 500 tokens were usually outliers, often reflecting non-standard abstract formats that disproportionately increased noise. We empirically evaluated several token-length ranges, and the 30-500 token window consistently produced the most coherent and interpretable topic structures. A dictionary of tokens was generated using the Gensim library to map each unique token to an identifier. To further stabilize the vocabulary and improve topic coherence, we applied frequency-based token pruning. Tokens appearing in fewer than 5,000 documents were excluded because they were too sparse and tended to introduce highly specific but statistically insignificant terms that biased topic formation. Conversely, tokens present in more than 30% of documents were removed due to their non-discriminative nature. These empirically determined thresholds minimized noise and optimized topic coherence. Finally, the preprocessed documents were converted into a bag-of-words representation, capturing token frequencies for structured computational analysis.

### LDA topic modeling

LDA was employed to uncover latent topics within the preprocessed abstracts [13]. Coherence scores, which measure the interpretability and semantic consistency of topics, were calculated using the Gensim library’s CoherenceModel function. A range of potential topic numbers, from 5 to 50 topics in increments of 5, was evaluated. For each specified number of topics, an LDA model was trained on the bag-of-words representation of the dataset. The coherence score for each model was computed using the c_v metric, based on the agreement of word co-occurrences within topics. The number of topics with the highest coherence score (30) was selected as the optimal model configuration. Once the optimal number of topics was determined, a final LDA model was trained using 20 passes over the dataset to ensure convergence. The model was implemented using Gensim’s LdaModel function, with the number of topics set to 30 and the preprocessed dictionary as input.

To assess the interpretability and relevance of the resulting topics beyond quantitative coherence metrics, we manually examined the TF-IDF-derived top words associated with each topic to verify their alignment with well-established thematic areas in aging research. This review confirmed that the top terms were consistent with meaningful and recognizable domains within the field. As an additional validation step, we randomly selected 30 representative abstracts from the corpus and identified the dominant topic assigned to each. This analysis, provided as Supplementary Data 1, demonstrated strong alignment between the model-assigned topics and the actual content of individual abstracts, further supporting the interpretability of the topic structure. To facilitate the exploration and presentation of the LDA model, interactive visualization was performed using the pyLDAvis library [23].

### TF-IDF modeling

To further refine the characterization of topics generated by the LDA model, a TF-IDF model was applied [9]. TF-IDF assigns a weight to each term in a document, reflecting its relative importance across the corpus. The TF-IDF model was trained on the bag-of-words representation (bow_corpus) using Gensim’s TfidfModel. The resulting TF-IDF matrix provided a weighted representation of the corpus, where each word was assigned, a score based on its frequency in a document relative to its prevalence across all documents. For each document, the topic distribution was computed using the LDA model’s get_document_topics function. The TF-IDF scores were then aggregated for each word within each topic, weighted by the topic distribution scores for the corresponding documents. This allowed for the computation of differential word importance across topics, providing a more nuanced understanding of topic-specific vocabulary.

### Topic proportion evolution

To analyze the temporal dynamics of topics, the yearly proportions of topics were calculated based on the LDA model’s topic distributions across the corpus. A five-year moving average was applied to smooth the data, reducing short-term fluctuations while preserving long-term trends.

### Shannon entropy calculation

Shannon entropy was calculated to quantify the evolution in the diversity of topic distributions within individual documents. Topic probabilities for each document were extracted from the LDA model. Entropy values were computed using the entropy function from the scipy.stats module [24], which measures the variability in topic assignment. Higher entropy values indicate greater diversity in the topic proportions within a document. Entropy values were paired with their corresponding publication years, and the mean entropy was calculated for each year. A 10-year rolling average was applied to smooth the data.

### Topic co-occurrence

To analyze the relationships between topics, a topic co-occurrence matrix was constructed based on the top topic probabilities per document. For each document, the five topics with the highest probabilities were identified from the LDA model’s topic distribution. Co-occurrence was defined as the simultaneous presence of any two topics within this top-5 set. A symmetric co-occurrence matrix was created where each cell represented the number of documents in which two topics co-occurred. The matrix was normalized by the total number of documents to calculate relative co-occurrence frequencies. To visualize the co-occurrence patterns, D3Blocks library chord function was used with links representing topic pairs exceeding a threshold of 0.033.

### Sentiment analysis

Sentiment analysis was conducted to assess the emotional tone of abstracts and explore temporal sentiment dynamics. Sentiment scores were computed using the VADER (Valence Aware Dictionary and sEntiment Reasoner) tool from the nltk library [25]. For each abstract, a compound sentiment score was computed, providing a single measure of overall sentiment. Keywords were extracted from the corpus using the TF-IDF method, selecting a maximum of 500 features. The extracted keywords were used to investigate sentiment trends in the dataset. A dictionary was created to store the sentiment scores associated with each keyword and the average sentiment per keyword was computed. To examine sentiment trends over time, the dataset was grouped by publication year, and the average sentiment scores for each keyword were calculated annually.

### Documents UMAP embedding and clustering

Document embeddings were generated and clustered to identify patterns and groupings within the corpus. First, the preprocessed documents were transformed into a high-dimensional vector using TF-IDF vectorization, retaining a maximum of 500 features to focus on the most significant terms. PCA with 50 components was applied to the TF-IDF matrix, UMAP was then used to project the PCA-transformed data into a two-dimensional space. To identify discrete clusters, Leiden clustering was performed on PCA result. The clustering was implemented using the AnnData framework from the Scanpy library [10] with a 0.7 resolution.

We used a relatively low number of neighbors (5) and retained 50 PCs, which together explained approximately 30% of the variance. This parameter selection was based on empirical observations that higher numbers of neighbors or PCs led to over-smoothed and noisier clustering, diminishing the capture of semantically meaningful topics. In high-dimensional textual data such as scientific abstracts most of the variance captured by the top PCs often reflects general language structure or high-frequency but non-informative terms. Consequently, retaining a smaller number of components can better preserve domain-specific variation. Similarly, using fewer neighbors in graph-based community increases local sensitivity, allowing the algorithm to better distinguish between fine-grained thematic areas. In contrast, in our experience using a high neighbor count tended to blur boundaries between conceptually distinct clusters, particularly in corpora with overlapping vocabulary but divergent contextual usage.

To identify the most distinctive words for each cluster, documents were assigned to clusters based on Leiden clustering results, and the mean TF-IDF score for each term within each cluster was calculated. Differential scores were computed for each term by comparing its mean TF-IDF score in a cluster to the maximum mean TF-IDF score across other clusters, highlighting words that were most specific to each cluster. Each document was checked for the presence of 3 top words using regular expression matching. The proportion of documents within each cluster containing these words was calculated, normalizing by the total number of documents in that cluster.

### Cosine similarity

Cosine similarity was used to measure the semantic similarity between clusters based on their mean TF-IDF representations. Preprocessed documents were transformed into TF-IDF vectors with a maximum of 500 features to balance computational efficiency and representation of significant terms. Documents were assigned to clusters according to Leiden clustering results, and the mean TF-IDF vector was calculated for each cluster by averaging the TF-IDF vectors of all documents within the cluster. A cosine similarity matrix was computed between the mean TF-IDF vectors of all clusters, providing a pairwise measure of similarity.

### Topic enrichment and hierarchical clustering of documents

Topic enrichment analysis was performed to identify the overrepresentation of specific topics within document clusters. For each cluster, topic distributions were aggregated across all documents, and enrichment scores were calculated by comparing observed topic proportions to expected values under a null distribution. Expected values were computed based on the overall topic distribution across the entire dataset, accounting for the size of each cluster. Enrichment scores were calculated using the log2 ratio of observed to expected values, with adjustments to avoid division by zero. To visualize the relationships between clusters and topics, hierarchical clustering was employed based on Euclidean distance and average linkage, with enrichment scores normalized to enhance interpretability.

### Evolution of document clusters

The temporal evolution of document clusters was analyzed by examining their proportions over time. Documents were assigned to clusters using Leiden clustering results, and their corresponding publication years were extracted. The mean publication year for each cluster was calculated to capture the average temporal distribution of documents within that cluster. Additionally, for each year, the proportion of documents belonging to each cluster was computed by normalizing cluster counts by the total number of documents published that year. To smooth temporal trends, a five-year moving average of these yearly proportions was applied.

### Cosine similarity trend

The temporal trends in topic distributions within document clusters were analyzed using cosine similarity to quantify changes in thematic consistency over time. For each cluster, annual topic distributions were averaged across all documents, resulting in yearly cluster-specific topic vectors. Cosine similarity was calculated between consecutive years to measure the degree of similarity in topic distributions, with values closer to 1 indicating higher thematic stability. To visualize long-term trends, a five-year moving average of cosine similarity was calculated for each cluster. Additionally, linear regression was applied to the five-year moving averages to quantify the direction and magnitude of trends in cosine similarity for each cluster. Clusters were ranked based on the slopes of these trends, identifying whether thematic stability was increasing or decreasing over time.

### BoA-related documents UMAP embedding and clustering

Documents related to the BoA were identified by filtering documents whose dominant topics included specific themes related to molecular and cell biology, yielding a total of 96,696 documents. The dominant topic for each document was determined based on the highest-probability topic in the document’s topic distribution from the LDA model. Similarly to our previous approach with all the documents, the filtered documents were transformed into high-dimensional vectors using TF-IDF vectorization, retaining 500 features, reduced to 30 dimensions with PCA, and further embedded into a two-dimensional space using UMAP. Leiden clustering was applied to the PCA result to identify subgroups within the BoA-related documents with a resolution of 0.7 using Scanpy. Distinctive words for each cluster were identified by calculating differential scores based on mean TF-IDF values, highlighting terms most specific to each cluster. Finally, the proportion of documents containing the top three words was computed for each cluster, normalized by the total number of documents.

### Hallmarks of aging presence in BoA clusters

The presence of hallmarks of aging related terms was analyzed across clusters by examining the proportion of documents containing predefined stems. The analyzed stems included ‘oxid’, ‘inflamm’, ‘senesc’, ‘mitochondri’, ‘genom’, ‘stem’, ‘insulin’, ‘metabol’, ‘telomer’, ‘epigenet’, ‘autophagi’, ‘nutrient’, ‘microbi’, and ‘intercellular’. Documents were assigned to clusters based on Leiden clustering, and the presence of each stem was checked in each of the documents. The proportion of documents containing each term was calculated for every cluster.

### Evolution of BoA-related documents and cosine similarity trend

The temporal evolution of document clusters related to the BoA was analyzed as previously described for the entire dataset, by examining their mean publication year and proportions over time. Additionally, cosine similarity trend was also calculated following the same strategy as the one described for the whole dataset.

### Semantic overlap analysis between clusters using TF-IDF scoring

The relationships between clusters were explored by analyzing the distribution of top cluster-specific words across other clusters using TF-IDF scores, both in the whole dataset and in the BoA-focused. For each cluster, the mean TF-IDF vector was calculated by averaging the TF-IDF scores of all documents within the cluster. The top 20 words with the highest TF-IDF scores were identified for each cluster, representing the most distinctive terms. To quantify the representation of cluster-specific words in other clusters, the mean TF-IDF scores of the top words from one cluster were calculated across all other clusters. This analysis highlighted the cluster-specific vocabulary that was over or underrepresented in other clusters.

### Data and code availability

All the data needed to generate all the figures, as well as preprocessed data is available either in Supplementary Data or through Figshare: https://doi.org/10.6084/m9.figshare.c.7711070. An interactive visualization of the LDA model can be found at https://github.com/jsanzros/aging_literature. Analyses were carried out using Python 3.11.12 with Google Colab TPU as backend. All code used for the analysis is publicly available online (https://github.com/jsanzros/aging_literature).

## Supplementary Material

Supplementary Figures

Supplementary Table 1

Supplementary Table 2

Supplementary Table 3

Supplementary Table 4

Supplementary Data
