# Supplementary material for: A natural language processing–driven map of the aging research landscape
Source: Aging (Albany NY). 2025 Nov 25;17(11):2778–808. doi: 10.18632/aging.206340 (PMC12705180; doi:10.18632/aging.206340)
Supplement: Supplementary Figures [file aging-17-11-206340-s001.pdf]

SUPPLEMENTARY FIGURES

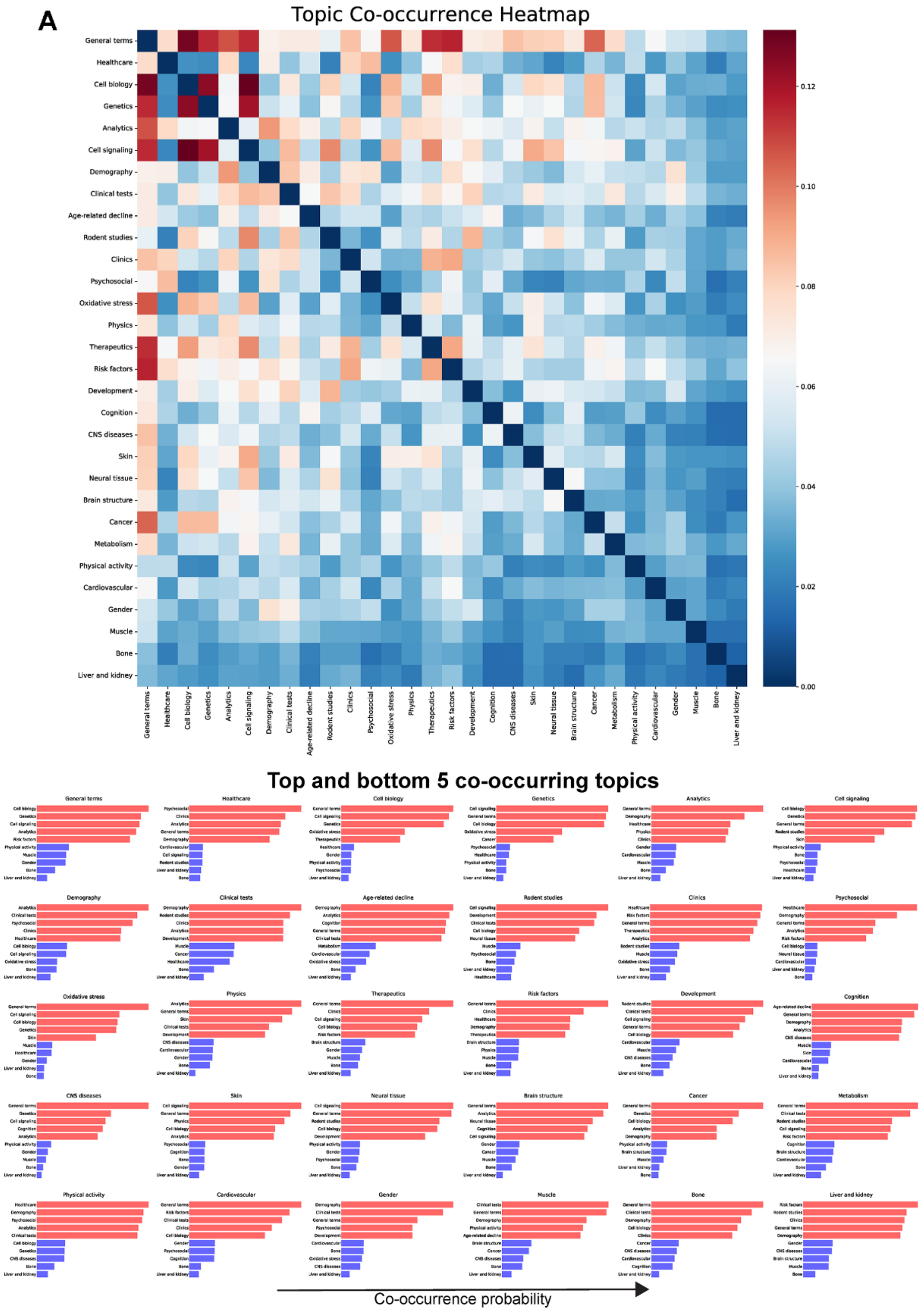

Supplementary Figure 1. Topic co-occurrence in aging research literature. (**A**) Heatmap of co-occurrence probability among all topics from LDA model with bar plots showcasing top and bottom 5 co-occurring topics.

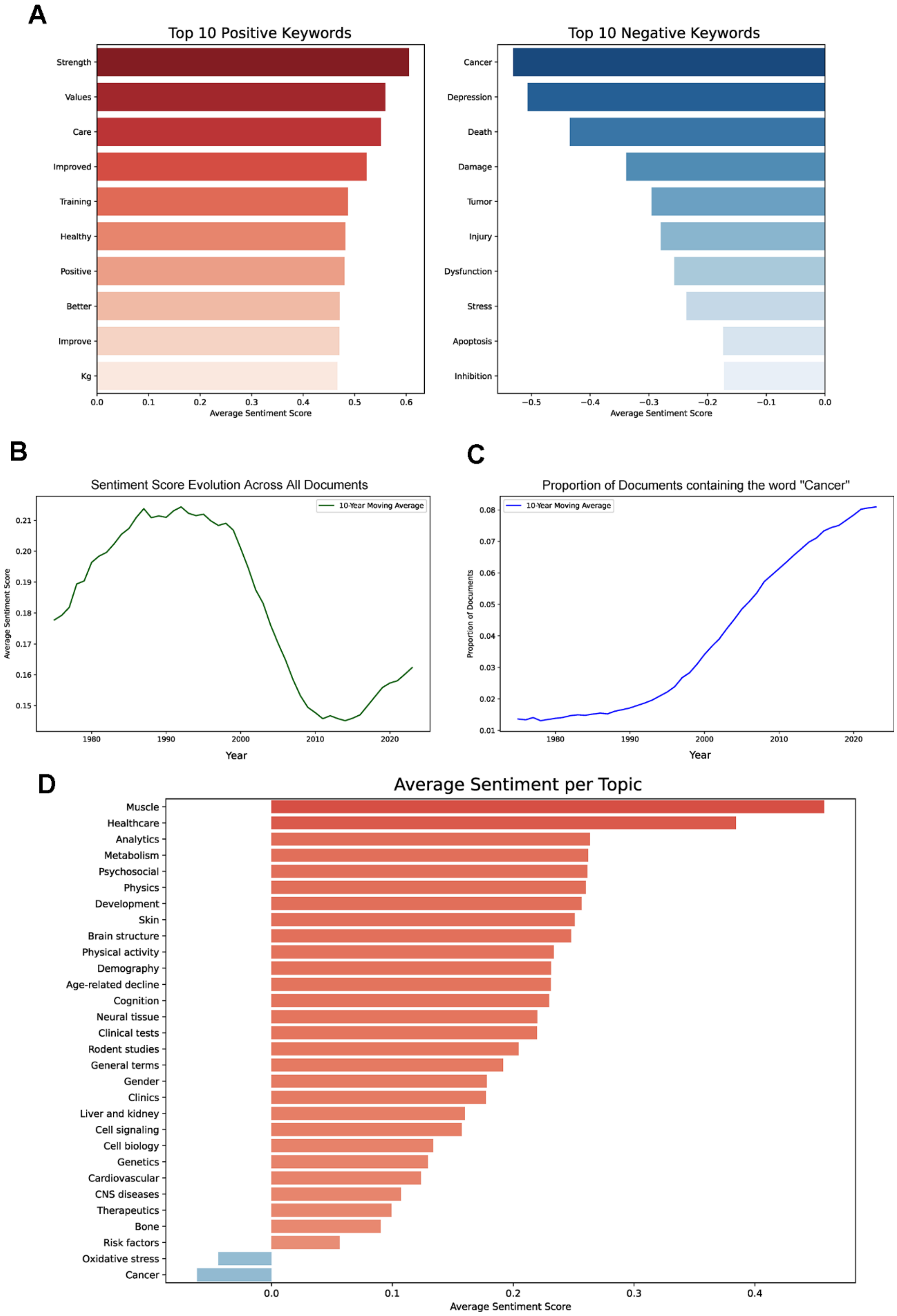

Supplementary Figure 2. Sentiment analysis of aging research literature. (**A**) Words with the highest and lowest sentiment score in all the corpus. (**B**) Evolution of sentiment score in the whole dataset. (**C**) Evolution of the proportion of documents containing the word cancer (word with the lowest sentiment score). (**D**) Average sentiment score per topic.

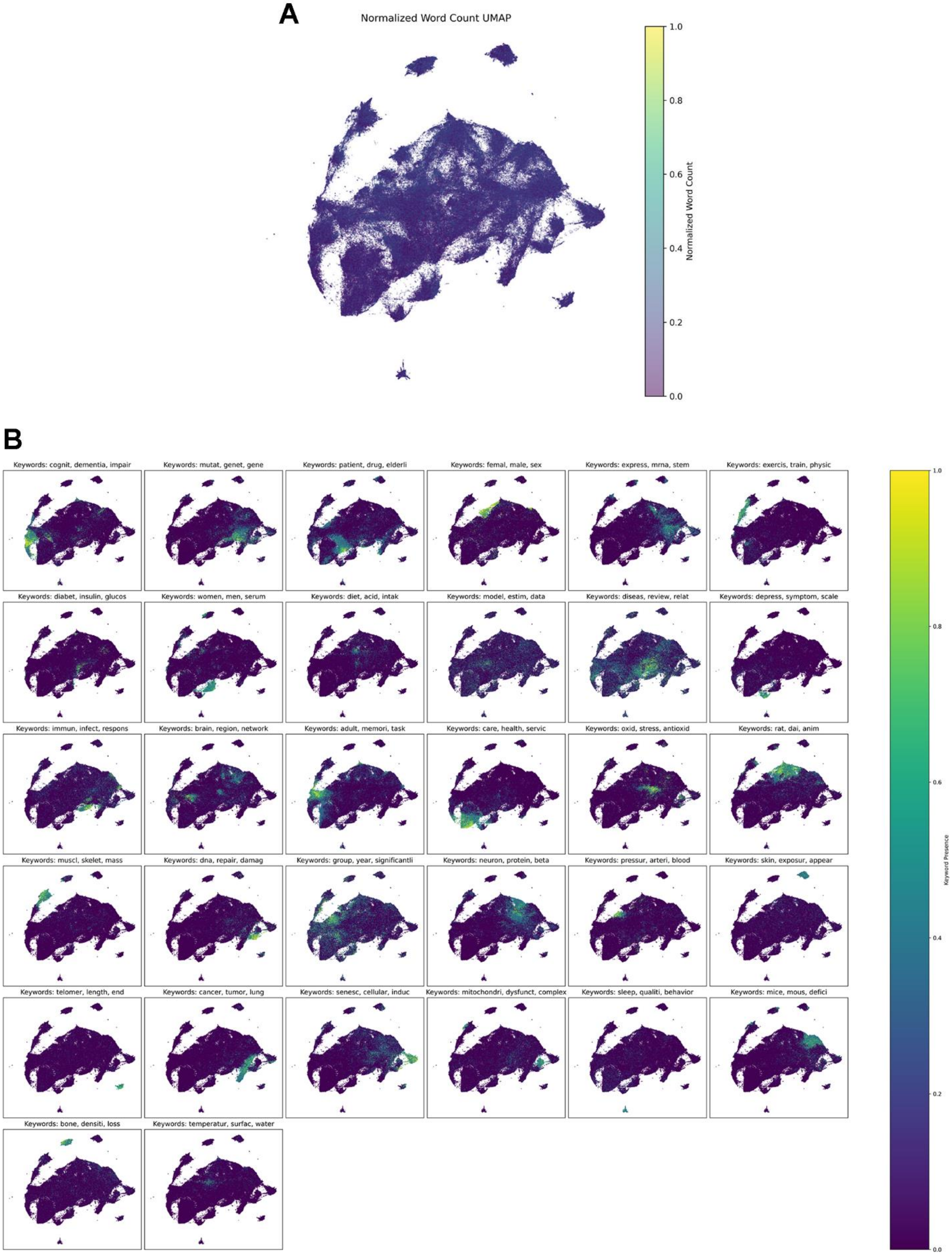

Supplementary Figure 3. UMAP visualizations (all documents). (**A**) Normalized word count per document. (**B**) UMAP visualization highlighting the presence of the three most distinctive words per cluster, identified using TF-IDF scoring.

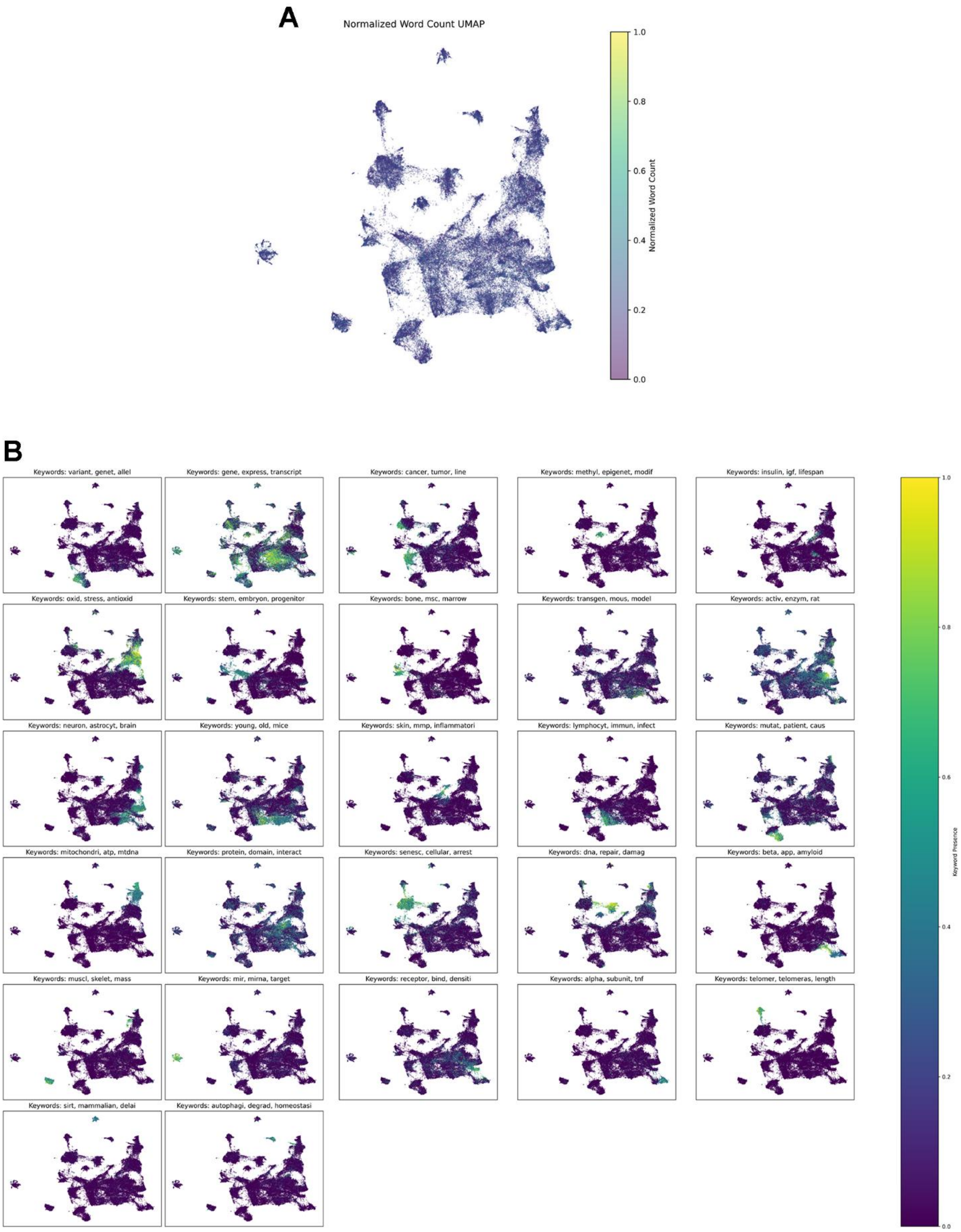

Supplementary Figure 4. UMAP visualizations (BoA documents). (**A**) Normalized word count per document. (**B**) UMAP visualization highlighting the presence of the three most distinctive words per BoA cluster, identified using TF-IDF scoring.
