# Supplementary material for: A natural language processing–driven map of the aging research landscape
Source: Aging (Albany NY). 2025 Nov 25;17(11):2778–808. doi: 10.18632/aging.206340 (PMC12705180; doi:10.18632/aging.206340)
Supplement: Supplementary Table 2 [file aging-17-11-206340-s003.docx]

Supplementary Table 2. Top 10 differential words between all clusters based on TF-IDF score.

| **Author_name** | **leiden** | **Top 1** | **Top 2** | **Top 3** | **Top 4** | **Top 5** | **Top 6** | **Top 7** | **Top 8** | **Top 9** | **Top 10** |
| --- | --- | --- | --- | --- | --- | --- | --- | --- | --- | --- | --- |
| Dementia | 0 | 'cognit' | 'dementia' | 'impair' | 'declin' | 'alzheim' | 'assess' | 'earli' | 'biomark' | 'better' | 'diagnosi' |
| Genetics | 1 | 'mutat' | 'genet' | 'gene' | 'associ' | 'identifi' | 'wide' | 'onset' | 'famili' | 'cohort' | 'case' |
| Geriatrics | 2 | 'patient' | 'drug' | 'elderli' | 'treatment' | 'clinic' | 'mortal' | 'stroke' | 'outcom' | 'therapi' | 'acut' |
| Gender | 3 | 'femal' | 'male' | 'sex' | 'longev' | 'select' | 'ratio' | 'collect' | 'rel' | 'number' | 'greater' |
| Cell signaling & stem cells | 4 | 'express' | 'mrna' | 'stem' | 'signal' | 'regul' | 'differenti' | 'receptor' | 'transcript' | 'deriv' | 'kinas' |
| Exercise | 5 | 'exercis' | 'train' | 'physic' | 'activ' | 'intervent' | 'improv' | 'program' | 'benefit' | 'post' | 'particip' |
| Diabetes | 6 | 'diabet' | 'insulin' | 'glucos' | 'obes' | 'type' | 'metabol' | 'resist' | 'sensit' | 'non' | 'preval' |
| Hormones | 7 | 'women' | 'men' | 'serum' | 'level' | 'hormon' | 'plasma' | 'statu' | 'ratio' | 'cross' | 'correl' |
| Nutrition | 8 | 'diet' | 'acid' | 'intak' | 'dietari' | 'food' | 'vitamin' | 'weight' | 'fat' | 'bodi' | 'restrict' |
| Statistics | 9 | 'model' | 'estim' | 'data' | 'rate' | 'standard' | 'variabl' | 'paramet' | 'predict' | 'propos' | 'describ' |
| Broad aging terminology | 10 | 'ag' | 'diseas' | 'review' | 'relat' | 'understand' | 'mechan' | 'discuss' | 'physiolog' | 'biolog' | 'process' |
| Depression & psychology | 11 | 'depress' | 'symptom' | 'scale' | 'relationship' | 'examin' | 'self' | 'major' | 'neg' | 'regress' | 'score' |
| Immunology | 12 | 'immun' | 'infect' | 'respons' | 'inflammatori' | 'inflamm' | 'adapt' | 'chronic' | 'specif' | 'consequ' | 'particularli' |
| Brain structure | 13 | 'brain' | 'region' | 'network' | 'connect' | 'imag' | 'cortex' | 'volum' | 'cortic' | 'area' | 'injuri' |
| Memory & learning | 14 | 'adult' | 'memori' | 'task' | 'older' | 'younger' | 'perform' | 'learn' | 'visual' | 'attent' | 'experi' |
| Healthcare | 15 | 'care' | 'health' | 'servic' | 'social' | 'home' | 'need' | 'peopl' | 'commun' | 'life' | 'live' |
| Oxidative stress | 16 | 'oxid' | 'stress' | 'antioxid' | 'anti' | 'free' | 'extract' | 'reactiv' | 'speci' | 'enzym' | 'oxygen' |
| Animal studies | 17 | 'rat' | 'dai' | 'anim' | 'month' | 'old' | 'decreas' | 'dose' | 'week' | 'administr' | 'increas' |
| Muscle | 18 | 'muscl' | 'skelet' | 'mass' | 'strength' | 'motor' | 'cross' | 'defin' | 'capac' | 'occur' | 'reduct' |
| DNA damage | 19 | 'dna' | 'repair' | 'damag' | 'site' | 'assai' | 'synthesi' | 'genom' | 'integr' | 'detect' | 'rna' |
| Comparative studies | 20 | 'group' | 'year' | 'significantli' | 'compar' | 'signific' | 'control' | 'children' | 'mean' | 'valu' | 'higher' |
| Alzheimer's | 21 | 'neuron' | 'protein' | 'beta' | 'tau' | 'amyloid' | 'alpha' | 'neurodegen' | 'local' | 'bind' | 'interact' |
| Vascular | 22 | 'pressur' | 'arteri' | 'blood' | 'hypertens' | 'vascular' | 'cardiovascular' | 'heart' | 'index' | 'measur' | 'reflect' |
| Skin | 23 | 'skin' | 'exposur' | 'appear' | 'treat' | 'techniqu' | 'effect' | 'tissu' | 'prevent' | 'evalu' | 'protect' |
| Telomeres | 24 | 'telomer' | 'length' | 'end' | 'short' | 'acceler' | 'maintain' | 'critic' | 'consequ' | 'rel' | 'link' |
| Cancer | 25 | 'cancer' | 'tumor' | 'lung' | 'surviv' | 'target' | 'incid' | 'overal' | 'line' | 'stage' | 'death' |
| Cell cycle & senescence | 26 | 'senesc' | 'cellular' | 'induc' | 'cycl' | 'prolifer' | 'suppress' | 'promot' | 'growth' | 'inhibit' | 'inhibitor' |
| Mitochondria | 27 | 'mitochondri' | 'dysfunct' | 'complex' | 'membran' | 'function' | 'gener' | 'accumul' | 'essenti' | 'link' | 'implic' |
| Sleep | 28 | 'sleep' | 'qualiti' | 'behavior' | 'pattern' | 'disord' | 'problem' | 'object' | 'particularli' | 'light' | 'consequ' |
| Mouse studies | 29 | 'mice' | 'mous' | 'defici' | 'strain' | 'reduc' | 'suggest' | 'demonstr' | 'elev' | 'abnorm' | 'indic' |
| Bone | 30 | 'bone' | 'densiti' | 'loss' | 'format' | 'tissu' | 'cross' | 'remain' | 'establish' | 'prevent' | 'occur' |
| Physics | 31 | 'temperatur' | 'surfac' | 'water' | 'properti' | 'phase' | 'time' | 'composit' | 'sampl' | 'degrad' | 'degre' |
