# Supplementary material for: A natural language processing–driven map of the aging research landscape
Source: Aging (Albany NY). 2025 Nov 25;17(11):2778–808. doi: 10.18632/aging.206340 (PMC12705180; doi:10.18632/aging.206340)
Supplement: Supplementary Table 3 [file aging-17-11-206340-s004.docx]

Supplementary Table 3. Top 10 differential words between BoA clusters based on TF-IDF score.

| name | leiden | Top 1 | Top 2 | Top 3 | Top 4 | Top 5 | Top 6 | Top 7 | Top 8 | Top 9 | Top 10 |
| --- | --- | --- | --- | --- | --- | --- | --- | --- | --- | --- | --- |
| GWAS | 0 | 'variant' | 'genet' | 'allel' | 'risk' | 'associ' | 'studi' | 'identifi' | 'genom' | 'wide' | 'signific' |
| Gene regulation | 1 | 'gene' | 'express' | 'mrna' | 'transcript' | 'pcr' | 'level' | 'development' | 'differ' | 'analysi' | 'blot' |
| Cancer | 2 | 'cancer' | 'tumor' | 'line' | 'apoptosi' | 'drug' | 'surviv' | 'apoptot' | 'lung' | 'high' | 'progress' |
| Epigenetics | 3 | 'methyl' | 'epigenet' | 'modif' | 'chang' | 'biolog' | 'alter' | 'acceler' | 'ag' | 'region' | 'pattern' |
| Insulin/IGF pathway | 4 | 'insulin' | 'igf' | 'lifespan' | 'elegan' | 'longev' | 'extend' | 'signal' | 'like' | 'growth' | 'life' |
| Oxidative stress | 5 | 'oxid' | 'stress' | 'antioxid' | 'radic' | 'peroxid' | 'free' | 'lipid' | 'sod' | 'reactiv' | 'oxygen' |
| Stem cells | 6 | 'stem' | 'cell' | 'progenitor' | 'tissu' | embryon' | 'adult' | 'differenti' | 'regener' | 'organ' | 'support' |
| Mesenchymal stem cells | 7 | 'bone' | 'msc' | 'marrow' | 'mesenchym' | 'format' | 'vitro' | 'potenti' | 'vivo' | 'capac' | 'deriv' |
| Mouse models | 8 | 'transgen' | 'mous' | 'model' | 'wild' | 'cognit' | 'defici' | 'patholog' | 'exhibit' | 'behavior' | 'femal' |
| Biochemistry | 9 | 'activ' | 'enzym' | 'rat' | 'decreas' | 'increas' | 'plasma' | 'liver' | 'measur' | 'concentr' | 'depend' |
| Neuroscience | 10 | 'neuron' | 'astrocyt' | 'brain' | 'hippocamp' | 'central' | 'death' | 'synapt' | 'neurodegen' | 'degener' | 'channel' |
| Comparative studies | 11 | 'young' | 'old' | 'mice' | 'respons' | 'produc' | 'week' | 'number' | 'deplet' | 'declin' | 'strain' |
| Skin | 12 | 'skin' | 'mmp' | 'inflammatori' | 'fibroblast' | 'inflamm' | 'matrix' | 'induc' | 'endotheli' | 'pro' | 'effect' |
| Immunology | 13 | 'elderli' | 'immun' | 'infect' | 'lymphocyt' | 'peripher' | 'older' | 'donor' | 'year' | 'cultur' | 'memori' |
| Clinical genetics | 14 | 'mutat' | 'patient' | 'caus' | 'famili' | 'syndrom' | 'disord' | 'featur' | 'encod' | 'character' | 'report' |
| Mitochondria | 15 | 'mitochondri' | 'mitochondria' | 'mtdna' | 'dysfunct' | 'atp' | 'energi' | 'chain' | 'function' | 'delet' | 'complex' |
| Protein biology | 16 | 'protein' | 'domain' | 'interact' | 'kinas' | 'phosphoryl' | 'translat' | 'contain' | 'local' | 'termin' | 'structur' |
| Senescence | 17 | 'senesc' | 'cellular' | 'arrest' | 'cycl' | 'induct' | 'transform' | 'trigger' | 'contribut' | 'stain' | 'accompani' |
| Genomic stability | 18 | 'dna' | 'repair' | 'damag' | 'doubl' | 'base' | 'effici' | 'replic' | 'integr' | 'agent' | 'stabil' |
| Alzheimer's | 19 | 'beta' | 'app' | 'amyloid' | 'tgf' | 'peptid' | 'precursor' | 'alzheim' | 'diabet' | 'shown' | 'possibl' |
| Muscle | 20 | 'muscl' | 'skelet' | 'mass' | 'synthesi' | 'content' | 'bodi' | 'improv' | 'understood' | 'follow' | 'acut' |
| RNA biology | 21 | 'mir' | 'mirna' | 'target' | 'downregul' | 'overexpress' | 'upregul' | 'rna' | 'assai' | 'knockdown' | 'confirm' |
| Receptor biology | 22 | 'receptor' | 'bind' | 'densiti' | 'cortex' | 'action' | 'select' | 'mediat' | 'block' | 'postnat' | 'modul' |
| Cytokines | 23 | 'alpha' | 'subunit' | 'tnf' | 'gamma' | 'contrast' | 'rel' | 'appear' | 'consist' | 'reduct' | 'possibl' |
| Telomeres | 24 | 'telomer' | 'telomeras' | 'length' | 'mainten' | 'chromosom' | 'human' | 'revers' | 'limit' | 'critic' | 'normal' |
| Sirtuins & mTOR | 25 | 'sirt' | 'mammalian' | 'delai' | 'improv' | 'oocyt' | 'subsequ' | 'kei' | 'unknown' | 'plai' | 'underli' |
| Autophagy | 26 | 'autophagi' | 'degrad' | 'homeostasi' | 'intracellular' | 'process' | 'accumul' | 'mechan' | 'essenti' | 'role' | 'enhanc' |
