# Supplementary material for: A natural language processing–driven map of the aging research landscape
Source: Aging (Albany NY). 2025 Nov 25;17(11):2778–808. doi: 10.18632/aging.206340 (PMC12705180; doi:10.18632/aging.206340)
Supplement: Supplementary Data [file aging-17-11-206340-s006.pdf]

SUPPLEMENTARY DATA

Supplementary Data 1. Representative abstracts for each LDA topic.

Dominant topic: general terms

The recent research on ageing processes in mammals throws new light on the biochemistry of circadian clock. The already known regulatory pathways for biological rhythms and metabolism, combined with newly discovered functions of sirtuins, unveil a perspective for new hypotheses, regarding possible links between ageing and circadian rhythms. The NAD World hypothesis - postulated as a systemic regulatory network for the metabolism and ageing, linked with mammalian, NAD+ dependent Sirtuin 1 - conceptually involves two critical elements. One is the systemic, Nampt-controlled NAD+ (nicotinamide phosphoribosyltransferase) biosynthesis, where Nampt (nicotinamide phosphoribosyltransferase) acts as "propulsion" for metabolism and the other is NAD+ dependent deacetylase (SIRT1) - a regulator responsible for various biological effects, depending on its localisation in organism. In this approach, the role of sirtuins, which are evolutionary conservative, NAD+ dependent histone deacetylases, may be very important for the mammalian metabolic clock. This paper is a review of current research on possible links among SIRT1 (Sirtuin 1), metabolism and ageing with particular consideration of the NAD World hypothesis.

Dominant topic: healthcare

NHS continuing healthcare is a package of care that is arranged and funded solely by the NHS where an individual has been found to have a 'primary health need'. Individuals who may be eligible have a right to be assessed for NHS continuing healthcare, and this assessment is undertaken by healthcare professionals using a national framework. However, there is a lack of literature on continuing healthcare and its assessment process. The aim was to review the literature on undertaking and providing continuing healthcare in terms of workforce roles, education and training, and competencies. A literature search was undertaken to identify relevant literature on continuing healthcare. Primary searching of electronic internal databases and indices at the Royal College of Nursing and King's College London was used, alongside a further database search and hand searching. A narrative synthesis of the literature was used to synthesise the findings, and a thematic analysis was undertaken to identify themes from the literature. The literature search identified 100 articles, of which 84 were excluded because they did not meet the inclusion criteria or provided insufficient details in the abstract. A total of 18 articles were included and examined in detail. Four themes were identified in the literature: complexity of care in transitioning care from hospital to home; different care models; importance of education of healthcare professionals; and role of continuing healthcare. Healthcare professionals - including nurses - should receive further training in caring for older people, especially in relation to continuing healthcare. Since there is an increasing ageing population, there is an increasing requirement for continuing healthcare, and thus further research examining all aspects of this care is required.

Dominant topic: cell biology

Most T lymphocytes, including regulatory T cells (Treg cells), differentiate in the thymus. The age-dependent involution of this organ leads to decreasing production of T cells. Here we found that the output of new Treg cells from the thymus decreased substantially more than that of conventional T cells. Peripheral mouse and human Treg cells recirculated back to the thymus, where they constituted a large proportion of the pool of Treg cells and displayed an activated and differentiated phenotype. In the thymus, the recirculating cells exerted their regulatory function by inhibiting interleukin 2 (IL-2)-dependent de novo differentiation of Treg cells. Thus, Treg cell development is controlled by a negative feedback loop in which mature progeny cells return to the thymus and restrain development of precursors of Treg cells.

Dominant topic: genetics

Age-dependent changes in gene expression profiles were studied in vegetative Pinus radiata buds by means of differential display. Among several candidate cDNAs, a 327 bp fragment that shows high homology with an Arabidopsis thaliana 20S proteasome ATPase designated RPT5a was found. Northern hybridization confirmed that the accumulation of this transcript increases with tree ageing, suggesting a possible role of this AAA-ATPase gene in development-related specific proteolysis.

Dominant topic: analytics

Harmonizing measures in order to conduct pooled data analyses has become a scientific priority in aging research. Retrospective harmonization where different studies lack common measures of comparable constructs presents a major challenge. This study compared

different approaches to harmonization with a crosswalk sample who completed multiple versions of the measures to be harmonized.

Dominant topic: cell signaling

The efflux of K+ from aged human erythrocytes is regulated by Ca2+: 5--100 mumol/l Ca2+ stimulate and 1--5 mmol/l Ca2+ inhibit K+-efflux. In fresh erythrocytes Ca2+ is virtually without effect on transport of K+. 3,7-Dimethyl-1-(5-oxohexyl)-xanthine (pentoxifylline) (5.5 mmol/l) decreases the K+-efflux from fresh erythrocytes by 10%. Similar effect of pentoxifylline is observed on the Ca2+-induced K+-efflux from aged erythrocytes. The ATPase-activity of a human erythrocyte membrane is stimulated in in vitro experiments by 1--100 mumol/l Ca2+. Increased concentration of Ca2+ (1--5 mmol/l), inhibits ATPase-activity. Pentoxifylline (0.5 mmol/l) modulates the effect of Ca2+ (conc. 1 mmol/l) on ATPase. It can be suggested that the rheological effect of pentoxifylline (possibly chelated with Ca2+) is caused by the decrease of Ca2+-caused K+-efflux and by the regulation of ATPase-activity of erythrocyte membrane.

Dominant topic: demography

Although research suggests racial/ethnic disparities in influenza vaccination and mortality rates, few studies have examined racial/ethnic trends among US adolescents. We used national cross-sectional data to determine (1) trends in influenza vaccination rates among non-Hispanic white (hereinafter, white), non-Hispanic black (hereinafter, black), and Hispanic adolescents over time and (2) whether influenza vaccination rates among adolescents varied by race/ethnicity.

Dominant topic: clinical tests

The age related changes on free plasma testosterone (T), dihydrotestosterone (DHT) and oestradiol (OE2) were determined in 82 healthy adult males. Forty-six subjects were between 22 and 61 years of age (group I), 36 between 67 and 93 years (group II). The percentage of free, non protein bound hormone was determined by equilibrium dialysis of undiluted plasma against isotonic phosphate buffer at 37 degrees C. Total hormone concentrations were measured by radioimmunoassay. The percentage of free T was 2.24% (median), 1.65-3.42 (95 percentiles) in group I and 1.65% (1.24-2.26) in group II. The percentage of free DHT decreased from 1.17% (0.80-2.03) in group I to 0.83% (0.52-1.55) in group II. The decrease in % free Oe2 was only very small: group I = 2.49% (2.13-2.96), ad group II = 2.31% (1.95-3.17). The fall in free T by 43.3% from 12.2 ng/100 ml (6.74-25.0) in group I to 6.90 ng/ml (3.57-10.6) in group II was twice as high as that of total T, which decreased on an average by 20.6%. Free DHT decreased by 25.8%: group I = 578 pg/100 ml (266-987), group II =429 pg/100 ml (168-723), while total DHT was not significantly different between the two groups (-1.9%). Free Oe2 was increased in old age: group I = 42.4 PG/100 ML (26.0-69.4), GROUP II = 55.7 PG/100 ML (35.8-118.9). The increase in free Oe2 by 31.4% was almost as high as that of the total Oe2 (46.9%).

Dominant topic: age-related decline

Differences between young and old adults in matching a motor response to arrival of a moving stimulus may be partially attributable to age group differences in eye movements. In Experiment 1 the eye movement patterns used by young and old adults during performance of a coincidence-anticipation task were recorded. Age group differences in preferred pattern were noted but did not appear to be linked to task performance level. In Experiment 2, eye tracking error was recorded for young adults and many of the old adults tested in Experiment 1. Age group differences were attributable to task performance rather than eye tracking error. Further, the preferred eye movement pattern did not appear to be stable over time among the subjects retested. The eye-movement reaction time of the old adults was significantly longer than that of the young adults, but this did not appear to hamper the ability of the old adults to distinguish the stimulus speeds. Little evidence was found for linking visual search to task performance error.

Dominant topic: rodent studies

The capacity of hpGRF (1-44) to induce release of GH in young (3-4 month old) versus old (19-21 month old) male rats was compared in vivo and in vitro. Injection of 1 ug/kg hpGRF to rats anesthetized with sodium pentobarbital increased plasma GH concentrations in young animals to greater than 1600 ng/ml within 5 min, and a dose of 10 ug/kg hpGRF increased GH levels to approximately 1900 ng/ml at 10 min. In old rats, plasma GH concentrations were increased after hpGRF but the increases were significantly lower than in the young rats at 5, 10, and 20 min after injection (P less than 0.01). The total GH released in old animals was calculated to be approximately 50% of that in young animals. In vitro release of GH from anterior pituitary slices incubated with hpGRF was also compared between young and old animals. After 20 min incubation with hpGRF (10(-9) or 10(-7) M) or vehicle, both young and old animals demonstrated a dose related and equivalent increase in GH release. These results indicate a reduced capacity of

hpGRF to stimulate GH release in vivo in aged as compared to young male rats. This decline is believed to be due to a hypothalamic influence on the anterior pituitary since the in vitro response of anterior pituitary tissue to hpGRF was similar in young and old rats. The reduced response to hpGRF in old male rats in vivo may be due to increased release of or enhanced sensitivity to somatostatin.

Dominant topic: clinics

Although great advances have been made in both radiological diagnosis and antibiotic therapy of microbial infections, the treatment of spinal infections remains a major clinical challenge. Many of the patients affected are referred to spinal units with long delays. The general population is ageing and the number of immunocompromised patients, as well as the number of operative procedures for spinal disorders are increasing. The aim of our study was to evaluate the clinical presentations of spinal infections, options for their diagnosis, indications for treatment and their risk factors and the results of surgery.

Dominant topic: psychosocial

The current study examined how a perceived neighborhood environment was associated with older adults’ walking activity and the experience of positive affect. Study sample comprised 10,700 older adults, aged 65+, sampled from the Health and Retirement Study 2014-2015 in the United States. Results indicated that neighborhood social cohesion was significantly predicting older adults’ walking and positive affect. It was also revealed that walking engagement significantly contributed to the measure of positive affect. However, perceived neighborhood physical disorder did not account for additional variance in walking and positive affect. Final structural model involved three latent factors-neighborhood social cohesion, walking, and positive affect-and the goodness-of-fit indices of the model indicated an acceptable fit to the sample data. Public health and physical activity intervention in the context of neighborhood environment should facilitate social integration and informal social support that the neighborhood creates.

Dominant topic: oxidative stress

Hypoxia and aging determine on mammalian cells a stress response which implies modified production of oxidants, reactive oxygen species or reactive nitrogen species at the mitochondrial level, interfering with cell-signaling proteins and inducing mitochondrial damage, apoptosis occurrence and functional consequences.

Dominant topic: physics

(2,3-Epoxypropoxy)propyltrimethoxy silane surface modified layered double hydroxides (KH560-LDHs) were prepared and used to improve the ultraviolet ageing resistance of asphalt. The results of X-ray photoelectron spectrometry (XPS) indicated that KH560 has been successfully grafted onto the surface of LDHs. The agglomeration of LDHs particles notably reduced after KH560 surface modification according to scanning electron microscopy (SEM), which implied that the KH560 surface modification was helpful to promote the dispersibility of LDHs in asphalt. Then, the influence of KH560-LDHs and LDHs on the physical and rheological properties of asphalt before and after UV ageing was thoroughly investigated. The storage stability test showed that the difference in softening point (Î”<i>S</i>) of LDHs modified asphalt decreased from 0.6 Â°C to 0.2 Â°C at an LDHs content of 1% after KH560 surface modification, and the tendency became more pronounced with the increase of LDH content, indicating that KH560 surface modification could improve the stability of LDHs in asphalt. After UV ageing, the viscous modulus (<i>G’’</i>) of asphalt significantly reduced, and correspondingly, the elastic modulus (<i>G’</i>) and rutting factor (<i>G</i>*/sin Î´) rapidly increased. Moreover, the asphaltene increased and the amount of “bee-like” structures of the asphalt decreased. Compared with LDHs, KH560-LDHs obviously restrained performance deterioration of the asphalt, and helped to relieve the variation of the chemical compositions and morphology of asphalt, which suggested that the improvement of KH560-LDHs on UV ageing resistance of asphalt was superior to LDHs.

Dominant topic: therapeutics

Background: Oral chemotherapy use is increasing due to new drug approvals as well as the convenience of the administration of oral drugs. This increased use also raises concern regarding drug-drug interactions (DDIs) with concomitantly administered drugs, resulting in loss of therapeutic effect, decreased tolerability, and/or increased toxicity.

Objective: The objective of this study was to review existing evidence of the clinical impact of DDIs with oral chemotherapeutic agents.

Methods: A comprehensive search of literature using PubMed was conducted in April 2018 for studies of DDIs associated with oral chemotherapy. Included studies were in English. We included randomized clinical trials, observational studies, and case reports evaluating a DDI between any oral chemotherapy drug

and any other drug. Included studies needed to have at least one outcome of clinical relevance potentially attributed to the DDI, for example, effects on survival or toxicity. The quality of the articles was determined using published metrics appropriate for the study design.

Results: There were 2626 studies identified in the initial search, of which 35 met all eligibility criteria. These included 15 retrospective cohort studies, 16 case reports or case series and four post hoc analyses of clinical trials. Among these, DDIs contributed to a statistically significant change in a clinical outcome in 12 studies. Eight of these studies evaluated overall survival and progression-free survival and found that the presence of the DDI was associated with reduced survival.

Conclusion: Our findings suggest that more real-world studies evaluating the association between oral chemotherapy DDIs and clinical outcomes are needed. The adverse clinical outcomes due to DDIs may be a reason for treatment failures and therapy discontinuation.

Dominant topic: risk factors

Diabetes is a growing public health concern, and animal models of this disease are necessary for a full understanding of disease pathogenesis, progression, clinical sequelae, and treatment options. In particular, nonhuman primate models of diabetes are important because of their close genetic relationship to humans. Although numerous Old World primate models have been described, few studies have examined the possibility of using New World monkeys as an animal model for this disease. Streptozotocin (STZ) is a common diabetogenic drug that selectively destroys beta cells after uptake via the GLUT2 glucose transporter. Induction of diabetes using STZ was attempted in common marmosets (Callithrix jacchus). These animals showed increases in blood glucose consistent with diabetes only at STZ doses markedly greater than those used in other primate species. Additionally, all animals showed pathological evidence of acute renal and liver toxicity secondary to the treatment. In a subsequent comparative study of various nonhuman primates, GLUT2 immunostaining in pancreatic islets was used as a marker for sensitivity to STZ. Immunostaining of islets from a variety of nonhuman primate species indicated a reduced expression of pancreatic GLUT2 in New compared with Old World monkeys; this finding explains their resistance to diabetic induction with STZ. Furthermore, there were age-dependent differences in GLUT2 expression, with aged and infant macaques showing reduced expression. We conclude that New World monkeys are an inappropriate model for diabetes induction with STZ and that, with all primate species, it is important to consider the animals' age before diabetic induction with STZ is attempted.

Dominant topic: development

The fertile period for natural mating in dogs extends from before ovulation until day 5 post ovulation (PO) and involves a delay in oocyte maturation until 2-3 days PO and viability of secondary oocytes for 48-60 h or more. Spermatozoa do not enter the uterus after vaginal insemination in late oestrus. Cervical closure appears to occur on average 5 days PO, but conception may occur following intrauterine artificial insemination (IUAI) up to 8 days PO. Therefore, the present study was conducted to clarify the duration of fertility of canine ova. Using IUAI at 6, 7, 8 and 9 days PO (n = 5 bitches each) conception rates were 100%, 71.4%, 37.5% and 0%, respectively, with an average litter resorption rate of 30.8%, and with mean litter sizes and times to delivery PO being 4.3 +/- 1.6 and 64.3 +/- 0.3 days, 4.0 +/- 1.4 and 66.3 +/- 0.4 days, and 2.5 and 68 days for IUAI at 6, 7 and 8 days, respectively. The high pregnancy rates with IUAI at 6 and 7 days PO confirm that many canine oocytes are fertile at 4-5 days after maturation. The high rate of resorption was presumably because of aging of ova or asynchrony between embryonic development and the intrauterine environment.

Dominant topic: cognition

Previous research suggests that everyday discrimination is associated with worse concomitant performance in several cognitive domains, as well as faster subsequent declines in episodic memory. This study aimed to extend knowledge on the specificity, durability, and mechanisms of associations between everyday discrimination and cognition by using a comprehensive neuropsychological battery and a longitudinal mediation design.

Dominant topic: CNS diseases

The head retraction reflex consists of a brief, involuntary extension of the neck on tapping the upper lip. This reflex was found to be present in 17.2% of patients with Parkinson's disease, but in only 4.9% of normal controls and 8% of cases of senile dementia. In addition there was a correlation between the severity of the Parkinson's disease and the presence of the reflex. This suggests that the reflex is specifically related to the Parkinson's disease process rather than to aging or the presence of dementia.

Dominant topic: skin

Age-related changes in the reducible cross-links of soluble and insoluble collagens from the human skin and aorta were evaluated. Histidinohydroxymerodemosine decreased with age up to the fifth decade and then remained constant in the insoluble skin collagen but could not be detected in the insoluble aorta collagen. Dihydroxylysinonorleucine could be observed, if in a small amount, in the insoluble aorta collagen but not in both the soluble and insoluble skin collagens. Hydroxylysinonorleucine seemed to be present in only a small amount, although coeluting unknown substances made it difficult to evaluate correctly. Two unknown components decreasing with age were found; the one in the insoluble skin collagen and the other in the insoluble aorta collagen. Difference in reducible cross-link was indicated between the skin and aorta collagens.

Dominant topic: neural tissue

This study of the fine structure of sciatic nerve branches in normal old cats provides evidence indicating that segmental demyelination may account, in part, for the significant decrease with age in the mean axonal conduction velocity in these hindlimb nerves. Fibers of different diameters exhibited focal abnormalities of their myelin sheath. Lipid-like droplets and granulo-vacuolar debris were present in distended portions of the inner adaxonal rim and in the outer cytoplasmic compartment of the Schwann cell. These inclusions extended into the cytoplasm of the paranodal myelin loops and clefts of Schmidt-Lantermann. There also occurred disruption of the axoglial junctions and separation of the myelin loops from the paranodal axolemma which widens the nodes of Ranvier. Complete disruption of one or more contiguous segments of the myelin sheath was produced by interlamellar splitting and ballooning along the major dense and intraperiod lines. Axonal degeneration occurred less frequently and was not present in all hindlimb nerves.

Dominant topic: brain structure

Increasing evidence suggests that retinal microvasculature may reflect global cerebral atrophy. However, little is known about the relation of retinal microvasculature with specific brain regions and brain networks. Therefore, we aimed to unravel the association of retinal microvasculature with gray matter changes and structural covariance network using a voxel-based morphometry (VBM) analysis.

Dominant topic: cancer

Cancer is an age-related disease, as incidence and mortality for most types of cancer increase with age. However, how molecular alterations in tumors differ among patients of different ages remains poorly understood. Recent studies have shed light on the age-associated molecular landscapes in cancer. Here, we summarize the main findings of these current studies, highlighting major differences in the genomic, transcriptomic, epigenetic, and immunological landscapes between cancer in younger and older patients. Importantly, some cancer driver genes are mutated more frequently in younger or older patients. We discuss the potential roles of aging-related processes in shaping these age-related differences in cancer. We further emphasize the remaining unsolved questions that could provide important insights that will have implications in personalized medicine.

Dominant topic: metabolism

Obesity and insulin resistance are associated with an impaired sensitivity to anabolic stimuli such as dietary protein (anabolic resistance). Omega-3 polyunsaturated fatty acids (n-3 PUFA) may be protective against the deleterious effects of saturated fatty acids (SFA) on insulin resistance. However, the contribution of excess fat consumption to anabolic and insulin resistance and the interaction between SFA and n-3 PUFA is not well studied.

Dominant topic: physical activity

Physical function is not routinely measured in older adults receiving dialysis. We evaluated the appropriateness of repeated measurements of physical function, including Short Physical Performance Battery (SPPB), handgrip strength, and activities of daily living (ADLs), in older adults receiving dialysis.

Dominant topic: cardiovascular

Age is associated with increases in elastic artery stiffness and pulse wave velocity, which cause profound changes in arterial pressure waves, including increases in the augmentation index (AIx) and wasted left ventricular (LV) energy. We examined the impact of aging on the central blood pressure (BP) waveform and wave reflection responses during exercise.

Dominant topic: gender

By establishing a statistical profile of the average sexual behaviour of women during the climacteric period, an attempt was made to determine what influence the climacteric had on female sexual activity. With the advancement of age, the sexual activity of women tends to decline. The menopause itself tends to further reduce the retrogression of the sexual activity in women.

Dominant topic: muscle

Aging of skeletal muscle is characterized not only by a decrease of muscle quantity but also by changes in muscle quality, such as an increase in muscle stiffness. The present study aimed to investigate the effects of supplementation with quercetin glycosides (QGs), well-known polyphenolic flavonoids, combined with resistance exercise on muscle quantity and stiffness.

Dominant topic: bone

The increase in bone fragility after menopause results from reduced periosteal bone formation and increased endocortical resorption. Women with highest remodeling had greatest loss of bone mass and estimated bone strength, whereas those with low remodeling lost less bone and maintained estimated bone strength.

Dominant topic: liver and kidney

The use of liver allografts from an older donor (OD) (age>50 years) is a widespread strategy to manage the disparity between supply and demand of organs for liver transplantation. This study determines the effect of OD allografts on fibrosis progression and graft survival after liver transplantation in patients with and without infection caused by hepatitis C virus (HCV).
